# Coevolution Theory of the Genetic Code at Age Forty: Pathway to Translation and Synthetic Life

**DOI:** 10.3390/life6010012

**Published:** 2016-03-16

**Authors:** J. Tze-Fei Wong, Siu-Kin Ng, Wai-Kin Mat, Taobo Hu, Hong Xue

**Affiliations:** Division of Life Science and Applied Genomics Center, Hong Kong University of Science & Technology, Clear Water Bay, Hong Kong; bcnskaa@hotmail.com (S.-K.N.); bcmwk@ust.hk (W.-K.M.); thuac@connect.ust.hk (T.H.); hxue@ust.hk (H.X.)

**Keywords:** gene, messenger RNA, transfer RNA, coevolution theory, Peptidated RNA World, last universal common ancestor (LUCA), intron, wobble

## Abstract

The origins of the components of genetic coding are examined in the present study. Genetic information arose from replicator induction by metabolite in accordance with the metabolic expansion law. Messenger RNA and transfer RNA stemmed from a template for binding the aminoacyl-RNA synthetase ribozymes employed to synthesize peptide prosthetic groups on RNAs in the Peptidated RNA World. Coevolution of the genetic code with amino acid biosynthesis generated tRNA paralogs that identify a last universal common ancestor (LUCA) of extant life close to *Methanopyrus*, which in turn points to archaeal tRNA introns as the most primitive introns and the anticodon usage of *Methanopyrus* as an ancient mode of wobble. The prediction of the coevolution theory of the genetic code that the code should be a mutable code has led to the isolation of optional and mandatory synthetic life forms with altered protein alphabets.

## 1. Introduction

The chain of information transfers from DNA to messenger RNA, and through genetic coding to proteins requires the assembly of multiple essential components. The pathway was a long one, winding through the ages to recruit all the components. Earlier we have enquired into the development of the gene, Peptidated RNA World, genetic code, last universal common ancestor (LUCA) and synthetic life. In the present study, enquiry is expanded to include the origins of messenger RNA, transfer RNA, intron, triplet codon, wobble base pairing and biological domains.

## 2. Origin of the Gene

Primitive Earth as a planet within a habitable zone was formed with chemical constituents including carbon compounds. Prebiotic chemical reactions on the planet together with an influx of matter from space produced building blocks including nucleotides and amino acids (aa). Accordingly, prebiotic chemistry was compatible with the rise of a living world based on nucleic acids and proteins as informational macromolecules. In this regard, the capability of RNA to serve both information storage and catalysis supports the formation of an RNA World prior to the present-day Protein World [1,2,3,4]. Abiotic synthesis of RNA endowed with prescriptive functional information, however, was obstructed by the twin pitfalls: First, prebiotic RNA production led to overwhelmingly useless random RNA sequences, such that RNA of the mass of the Earth had to be synthesized to yield two or more copies of a 40-mer self-replicating RNA to initiate abiotic RNA replication [5]; and, secondly, template-directed RNA replication gave rise to dead-end double-stranded complexes that could not be pulled apart to renew replication [6,7,8].

Although a range of physicochemical systems have been investigated with respect to their potential to generate informational macromolecule evolution, including chaos theory, complexity theory, fractals, rugged fitness landscapes, Markov chains, hypercycles, dissipative structures, Shannon information theory, autopoiesis, evolutionary algorithms and directed evolution, none of them can selectively give rise to enrichment of RNAs endowed with prescriptive functional information [9]. The only mechanism found to enrich functional RNAs (fRNA) over useless RNAs and overcome the twin pitfalls is *replicator induction by metabolite*, whereby dead-end duplexes containing fRNAs capable of binding metabolite ligands are selectively split apart by the ligands to restart template-directed polymerization. In contrast, dead-end duplexes containing non-functional RNAs that do not bind any metabolite ligand will remain unsplit and degrade, releasing their nucleotides for incorporation into the fRNAs [6,8]. The outcome is expressed by the metabolic expansion law:

Under conditions of active synthesis of RNA-like replicators, accelerated template-directed synthesis of RNA-like replicators, and the presence of a huge population of random RNA-like duplexes in the environment, functional RNA-like aptamers/ribozymes will be selectively amplified by their cognate metabolites in the environment through the replicator induction by metabolite (REIM) mechanism based on the metabolic expansion equation, leading to the appearance of novel RNA-like ribozymes catalytically acting on the metabolites to form novel metabolites and thereby expand metabolism.

In the metabolic expansion equation,

R = ∫ k α R (1 − R + σ) dt
(1)

R represents fRNA *viz.* aptamer or ribozyme, k the rate constant of template-directed fRNA synthesis, α the ratio between template and fRNA, and σ the influx of nucleotides from environment. The equation predicts a monotonic rise of fRNA to totally dominate over non-functional RNA, so that the entire pool of prebiotic random RNAs converts to functional RNAs, opening up an RNA World and life. Within the living system, the prescriptive sequence information in the functional RNAs is transformed to genetic information, and the functional RNAs become genes.

## 3. Origin of Messenger RNA

Prebiotic syntheses including ones brought about by hydrothermal vents [10,11], organic compounds in meteorites [12], and facilitation of RNA polymerization and template-directed replication by water-ice systems, lipid bilayers and mineral surfaces to yield trace quantities of fRNAs [13,14,15,16,17,18,19] could predispose the production of fRNA through REIM at favorable geological niches such as fire-and-ice reactor (FAIR) sites on prebiotic Earth with icy conditions and nearby hydrothermal vents [10,20]. This led to a *march of progress* from simple ribozymes to ribozyme assembly via tag sequences, and rudimentary replication by template-directed polymerase ribozyme to arrive at an RNA World [21]. However, ribozymes as biocatalysts can usefully achieve low Michaelis constants toward substrates, but their catalytic rate constants are far below those of enzymes [22,23].

In the modern Protein World, 20 genetically coded amino acids provide a well-balanced set of side chains to enable superb catalysis as exemplified by diffusion-controlled enzymic reactions, yet the *side chain imperative* is so strong that numerous amino acid side chains have been added to proteins through post-translational modifications. Primitive aptamers and ribozymes with only four types of nucleotide subunits experienced an even greater pressing need for additional side chains to serve their targeted functions. However, in the RNA World, increases in side chains could not be sought through adoption of more nucleotides in the RNA alphabet, for expansion to more than the U, C, A and G nucleotide letters would risk enhancing the scope of base pairing errors. Therefore, post-replication modification (PRM) represents the only assured access to side chain expansion, as indicated by the importance of wide ranging modified nucleosides to cellular RNAs even in the Protein World [24]. Given the presence of amino acids on prebiotic Earth, inevitably some PRMs involved the covalent attachment of amino acids and peptides to fRNA. In time, the catalytic prowess of the attached polypeptide prosthetic groups overshadowed that of the ribozymes themselves, and opened up the Peptidated RNA World [25] where these polypeptide prosthetic groups established numerous polypeptide folds and domains. Although in the beginning they performed their ligand binding, catalytic and structural tasks while attached to the host fRNAs, they later detached from the fRNAs to perform as primitive proteins. Thus, the two major steps in biocatalysis evolution were:

Ribozymes → Peptidated Ribozymes → Enzymes
(2)

In the first step, the peptide prosthetic group on fRNA evolved to cooperate with host fRNA, as illustrated by known RNA-peptide interactions where modest-sized peptides can strongly affect the structure and function of fRNA [26,27,28]. The second step is illustrated by the displacement of rRNA stem structures by r-proteins in human mitochondrial ribosomes, and fRNA by protein in chloroplast signal recognition particle [29,30].

In the face of mounting evidence for the inadequacy of RNA acting alone without peptides, there is growing consensus that RNA-protein collaboration was crucial to the development of present-day life [28,31,32,33,34,35], which is attested to by the finding that ribosome evolution stemmed not from RNA alone but from interactions between the oldest rRNA and r-protein sequences long before the arrival of a mature ribosomal peptidyl transferase center (PTC) for protein synthesis [36,37]. However, as emphasized by the *information-need paradox*, biopolymers that might fulfill the roles of informational macromolecules are too long to be able to arise spontaneously [38]. For information-rich fRNAs, this obstacle is readily overcome by REIM. For proteins, on the other hand, there was no selective mechanism comparable to REIM, and therefore no workable pathway to conjure up an array of information-rich functional proteins from amino acids. In contrast, with peptidated fRNAs, their protein prosthetic groups were at least in the beginning only functional add-ons. While any useful polypeptide prosthetic group was a beneficial advance, a useless polypeptide prosthetic group, like an unskilled apprentice, was of no value yet undamaging and tolerable. Accordingly, the polypeptide prosthetic groups covalently attached to fRNAs enjoyed the necessary freedom to explore protein sequence spaces through evolution to eventually arrive at useful protein folds, protein domains and ultimately detached, free-standing proteins/enzymes. It follows that the partners in the earliest RNA-protein collaborations could not be fRNAs and self-originated free-standing proteins. Instead, they were fRNAs and their covalently attached protein prosthetic groups. Through this uniquely accommodating nurture conferred by host fRNAs on their appended peptides, a trailblazing RNA World gave rise to a Peptidated RNA World, which in turn provided an ideal incubator for useful protein sequences for the Protein World.

How to determine the amino acid sequences of the peptide prosthetic groups of fRNAs was a major challenge for the Peptidated RNA World. Since each fRNA was its own gene, it would be cumbersome if it must look to other genes for a template to direct the building of its own PRM. Instead, each fRNA would evolve its own peptide-directing template for the PRM. A PRM was equivalent to a modern post-transcriptional modification (PTM) introduced into RNA, often by multiple RNA-modifying enzymes as in the synthesis of mnm^5^s^2^U34 and queuine on modern tRNA [39]. Moreover, because all enzyme reactions regardless of mechanism require complex formation between catalyst and substrate [40], a succession of specific aminoacylating enzymes must bind to the RNA substrate to produce a peptide PRM. On this basis, to build a tripeptide side chain such as Leu–Ser–Asp on an fRNA, expectedly there had to be a template on the fRNA for binding the aminoacyl-RNA synthetase ribozymes (rARS) for Leu, Ser and Asp, equipped with orderly arranged “codonic sites” for rLeuRS, rSerRS and rAspRS to ensure a Leu–Ser–Asp peptide sequence.

Accordingly, a template for binding rARS is an evident prerequisite to the building of a peptide PRM with a predetermined amino acid sequence. However, because RNAs are capable of binding amino acids, it has been suggested that the earliest template for peptide building comprised a direct RNA template for binding amino acids [41]. In addition, since modern messenger RNA as a peptide template binds tRNAs as intermediate amino acid acceptors rather than enzymes or amino acids, the possibility of the earliest peptide-directing template being a template for intermediate amino acid acceptors merits consideration along with the other two potential types of templates.

### 3.1. Self-rARS Template (SART)

**Figure 1:**
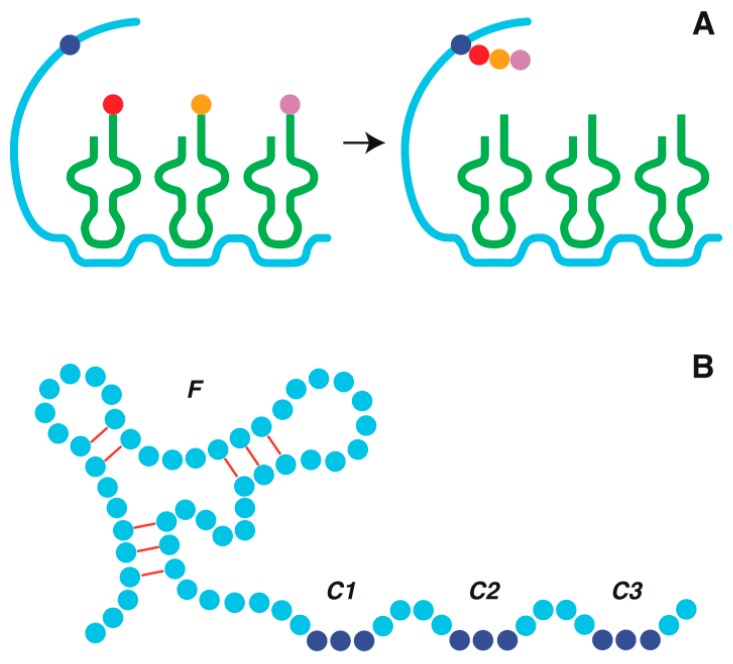
SART model of primitive messenger RNA. (**A**) Synthesis of a Leu–Ser–Asp side chain on target nucleotide X (dark blue circle) from Leu–rLeuRS, Ser–rSerRS and Asp–rAspRS conjugates (with Leu represented by purple, Ser by orange and Asp by red circles) bound to amino acid-specific codonic sites on the SART template. Peptide sequence correlates with order of codonic sites on template. Elongation of the peptide may proceed in N → C direction, which entails initial transfer of Leu to Ser–rSerRS, followed by Leu–Ser to Asp–rAspRS, and finally Leu–Ser–Asp to X; or in C → N direction, which entails initial transfer of Asp which is sited closest to X, followed by Ser, and finally Leu from their aa–rARS conjugates to X or growing peptide on X. Both the N → C and C → N modes are workable for short peptides. For long polypeptides, however, the amino acids at the N-terminus that add to the growing peptide last in the C → N mode would be distant from X and the growing peptide on X, rendering their additions problematic. Therefore, the N → C elongation mode was adopted for RNA peptidation, determining thereby also the N → C direction of peptide elongation in modern ribosomal protein synthesis, which differs from RNA peptidation only with respect to its omission of the final transfer of the completed peptide to X. (**B**) Primitive two-domain fRNA–mRNA as precursor of modern messenger RNA. F, functional aptamer/ribozyme domain; C1–C3, template domain with three triplet codons (dark blue circles).

The isolations of rARS achieved by a number of laboratories [42,43,44,45,46,47,48,49,50,51,52] yielded mostly self-aminoacylating rARSs with varied structures and amino acid specificities, suggesting that such self-rARSs were by no means rare occurrences in the RNA World. These self-rARSs display a range of diverse activities. Besides catalysis of cis-aminoacylation of their own 2′(3′)-OH, 5′-OH or internal 2′-OH, some of them are capable of trans-aminoacylation of RNA substrate, or cis-aminoacylation followed by trans-aminoacylation to yield aminoacyl or peptidyl ester, even via thioester linkages. The aa-rARS conjugates formed by self-rARSs through cis-aminoacylation could bind to codonic sites on a self-rARS template on an fRNA, and incorporate their aminoacyl moieties into a peptide PRM on the fRNA utilizing either their own trans-aminoacylation activities or a specialized trans-aminoacylator rARS, e.g., a miniscule five nucleotide rARS that works with different amino acids [51]. Either way, the order by which the codonic sites for different self-rARSs are arranged on the template would dictate the amino acid sequence of the resultant peptide PRM (Figure 1A).

### 3.2. Direct RNA Template (DRT)

The suggestion that a DRT could bind amino acids directly is supported by exhaustive searches revealing the binding of amino acids, including prebiotically available Leu and Ile to triplet codons [53]. However, the number of triplet codons capable of binding prebiotically available amino acids is limited, rendering it difficult for primitive encoding to be based solely on triplet codons. This is in accord with the ability of RNA to bind nucleosides and heterocycles well but less so with other classes of molecules [54]. Therefore, to proceed, DRT might not be able to rely on triplet codons alone. Anticodon-amino acid interactions have been suggested to play a significant role in this regard [55]. More complex multi-stranded RNA sites for amino acid binding also might be employed in the beginning, exemplified by Gln binding site on AD02 GlnRS ribozyme, Phe binding site on r24 PheRS ribozyme, Gly and Lys binding sites on their respective riboswitches, and the RNA-hairpin Arg binding site on the human HIV-1 messenger RNA TAR structure [49,52,56,57,58].

### 3.3. Intermediate Acceptor Template (IMAT)

In principle, any RNA sequence can serve as an intermediate amino acid acceptor, although the presence of an aminoacylatable 3′(2′)-OH could facilitate eventual transition of the acceptor to tRNA. In particular, the findings that acceptor stem-related partial structures of tRNA can be aminoacylated by aminoacyl-tRNA synthetase enzymes (eARS) [59,60,61] underline the possibility of such partial structures, e.g., loop-deficient tRNA, minihelix, microhelix, RNA tetraloop, *etc.*, acting as early amino acid acceptor ligands binding to an intermediate acceptor template on fRNA. Other tRNA partial structures that might serve in this capacity include half-tRNA molecules [62,63] and anticodon-containing coding coenzyme handles [64].

The three potential peptide-directing templates, *viz.* SART, DRT and IMAT, have different requirements with regard to their subsequent evolution into modern messenger RNA. In the case of DRT, this evolution process must satisfy three essential requirements:

(I) finding a cognate RNA acceptor for each amino acid to be employed in the peptide prosthetic groups on fRNAs;
(II) finding a cognate rARS to join each amino acid to its cognate RNA acceptor; and
(III) switching the original binding sites on the template designed for amino acids to binding sites for RNA acceptors of amino acids.

The need to satisfy Requirements I-III was burdensome for DRT. For IMAT, Requirement III was rendered unnecessary because there was no need to switch from amino acid binding to acceptor binding, leaving only Requirements I and II. For SART, Requirement II was also unnecessary because the RNA acceptor in Requirement I was also the rARS needed in Requirement II. Since Requirements II and III applied to all the amino acids for incorporation into peptide prosthetic groups, they amounted to substantial impediments. Accordingly, free of these impediments, SART not only played an essential role in organizing the multi-ribozymic synthesis of peptide PRMs, but also easily outpaced DRT and IMAT in its development to become the adopted template for directing peptide sequences in RNA peptidation. Acting as amino acid-accepting RNA ligands of SART, the self-rARSs were functionally equivalent to primitive self-charging tRNAs, more versatile than modern tRNAs on account of their catalytic capability and a remarkable invention of nature for peptide building. Given the availability of these self-charging tRNAs in the Peptidated RNA World, any fRNA could start constructing its own peptide prosthetic group simply by evolving a SART. Eons later, entering the Protein World, most ribozymes gave up their catalytic roles to their erstwhile polypeptide prosthetic groups and turn into mRNAs to encode the enzymes derived from such prosthetic groups. The self-charging tRNAs were no exception in this regard: They gave up their catalytic roles to their erstwhile eARS domains, and became tRNAs.

In developing sequences to house the codon sites for SART, it would be important for an fRNA not to perturb its catalytic/ligand binding sites by a scattered insertion of codon sites for self-rARS binding. Accordingly, sooner or later, the evolution of two different domains on an fRNA–mRNA would be favored to separately house the catalytic/ligand binding and peptide-encoding activities of the molecule, as illustrated in Figure 1B. Eventually, as the ribozyme/aptamer activity of fRNA was ceded to its polypeptide prosthetic group, the fRNA–mRNA was transformed into a two-domain messenger RNA prior to further transformation into a single purpose mRNA [8]. Today, relics of ancient two-domain mRNAs are found as riboswitches containing an aptamer domain that, upon binding a metabolite ligand, modulates the activity of a messenger domain [56,57].

Furthermore, examples of full-fledged two-domain fRNA–mRNAs persist among the catalytic introns. Group I introns including yeast mtDNA intron aI4α, *Chlamydomonas reinhardtii* ctDNA LSU intron, phage T4 thymidylate synthase (td) intron, bacteriophage SPO1 DNA polymerase I intron and *Physarum polycephalum* LSU intron 3 encode different types of associated DNA endonucleases. Since these DNA endonucleases function in intron mobility or splicing, they complement the Group I introns functionally, just as protein prosthetic groups would complement fRNAs in the Peptidated RNA World. Likewise, the best studied mobile Group II introns, *viz.* yeast mtDNA introns aI1 and aI2 in the *cox1* gene, and *Lactococcus lactis ltrB* intron in the putative relaxase gene of a conjugative element, all contain lengthy ORFs for 509–778 aa that encode proteins with reverse transcriptase (RT) activity adjacent to a putative maturase component [65]. It would be no surprise if such RTs date back to the transition of RNA genes to DNA genes. In addition, the numerous examples of ORF-less Group II introns that contain ORF remnants suggest that most ORF-less introns are derived from ORF-containing introns [66], which points to the antiquity of fRNA–mRNAs in support of their general occurrence in the Peptidated RNA World.

## 4. Origin of Transfer RNA

Since peptidation of fRNA, not ribosomal protein synthesis, was the origin of peptide coding, the developments of mRNA and tRNA took place within the Peptidated RNA World. The timelines for tRNA evolution show that the appearance of the tRNA acceptor stem was followed sequentially by the anticodon arm preceding the T arm in Archaea or the reverse in Bacteria and Eukarya, tRNA interaction with eARS domains, pre-PTC ribosome with SSU rRNA ratchet, tRNA D arm, tRNA variable arm and anticodon recognition by eARS domains, and post-PTC ribosome containing mature PTC [37,67,68].

**Figure 2:**
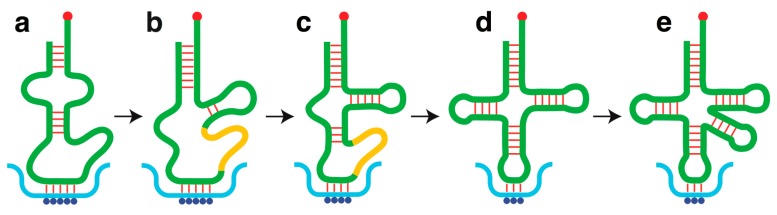
Development of transfer RNA. Stage a. Aminoacylated self-rARS serving as a self-charging tRNA is liganded to its cognate codonic site on SART. Stages b–e. Development of tRNA with successive additions of different substructures. Anticodon-codon pairing is in general more complex in the early stages than the late stages. Complementary base pairs and non-covalent bondings are represented by red lines, and amino acid esterified to 3′ terminus of evolving tRNA by red circle. Dark blue circles represent codonic bases on SART. Intron (orange band) is present in intermediate stages, and eliminated from some but not all of present-day tRNAs; its sequence could arise from a defunct segment of the self-rARS in Stage a, e.g., catalytic sequence functionally superseded by eARS, or its own former SART sequence the template function of which has been transferred to a paralog specialized as mRNA for eARS.

In the SART model, which postulates a ribozymic origin of tRNA, the earliest ligands bound by the SART template on fRNA were aa-rARS conjugates formed by self-rARSs. The self-rARSs isolated *in vitro* are highly heterogeneous, consisting variously of a bihelix, a helix-bulge-helix-loop, a cloverleaf-containing RNA, *etc.*, and with or without reported trans-aminoacylation activity. This suggests that the binding of the early aa-rARSs to codonic sites on SART would give rise at first to non-uniform *idiosyncratic peptidation* of fRNA akin to the idiosyncratic post-transcriptional modifications found on tRNA nowadays, which often vary between tRNAs or between organisms. Figure 2 shows different stages of tRNA development starting in Stage-a with an aminoacylated self-rARS acting as self-charging tRNA, followed in Stages b–e by the addition of tRNA substructures in keeping with the evolution timeline plus intron insertion at the intermediate stages. To enter into this development sequence, the self-charging tRNA had to be equipped with: (i) an amino acid-accepting hydroxyl group; (ii) an anticodon that paired with a complementary codon on SART; and (iii) a catalytic domain for cis-aminoacylation, and optionally also trans-aminoacylation. The successful self-charging tRNAs that morphed into tRNAs may be expected to leave behind ancestral imprints, such as a 3′-aminoacylatable acceptor stem, and possibly sequence bulges that could grow into tRNA arms, as illustrated by the self-charging tRNA shown in Stage-a, which possesses a helix-bulge-helix-loop structure comparable to a self-rPheRS that catalyzes *cis*-aminoacylation at its 2′(3′)-OH on an unstructured 3’ terminus [46].

Initially, the self-charging tRNAs might not need to be endowed with rigorous amino acid specificity to be useful. In particular, membrane transports are essential to living cells, yet hydrophilic RNAs do not associate well with lipid membranes. Accordingly, fRNA peptidation, even with limited discrimination between Val, Leu and Ile, could add hydrophobic transport peptide domains to fRNAs, converting them into membrane transporters. In view of the transporter-deficiency crisis bound to be faced by the earliest cells/precells in the RNA World, it is not surprising that the oldest protein structure detected by evolutionary timelines is linked to ATP binding cassette (ABC) transporters, which are universally distributed in the living world, constitute one of the largest protein families, and mediate the transport of a wide range of molecules across membranes [69].

Later on, when the catalytic self-charging tRNAs spinned off the SART segments encoding their prosthetic eARS protein domains and evolved into non-catalytic tRNAs, the various transfers of aminoacyl and peptidyl moieties between rARSs, and between rARSs and fRNA, also came to be catalyzed by peptidyl-transferase ribozymes (PTR) exemplified by clone-25 PTR [70], which contains structures similar to the PTC, including the central loop of domain V and the peptidyl-transferase loop of 23S rRNA. Upon the advent of PTR, idiosyncratic peptidation gave way to *centralized peptidation* by PTR at the pre-PTC ribosome [8]. Much later, when the development of mature PTC opened up the Protein World, pre-PTC ribosome was replaced by post-PTC ribosome, and PTR replaced by ribosomal PTC. Thus, the performance of centralized RNA peptidation at pre-PTC ribosome but modern translation at post-PTC ribosome could provide a possible rationale for the distinct pre-PTC and post-PTC ribosomes detected by evolutionary timelines [37].

The melting temperatures for RNA duplexes vary with duplex length, and triplet duplexes do not resist melting well at mesophile growing temperatures ranging up to 45 °C. That triplet codon-anticodon pairs between mRNA and tRNA can withstand mesophile, thermophile and even hyperthermophile growth temperatures is dependent on a 1000-fold enhanced association constant between codon and anticodon (relative to two linear complementary trinucleotides) arising from the loop constraint, dangling-end nucleotides flanking the anticodon and modified nucleotides in the dangling ends [71]. Accordingly, the self-charging tRNA is depicted in Stage-a binding to its codonic site on SART through more than three complementary base pairs. As tRNA development proceeded from Stage-a through to Stage-e with successive additions of tRNA substructures, optimizations of base sequence and nucleoside modifications in the anticodon loop would lead to a progressive enhancement of the codon-anticodon association constant, thereby enabling a reduction of the number of complementary base pairs between codon and anticodon (red lines) to arrive finally at a cloverleaf tRNA with a triplet anticodon.

In Stage-a, the specificity of the esterified amino acid was determined by the self-charging tRNA. As soon as its self-aminoacylating activity was replaced by that of an eARS domain or enzyme, charging of the tRNA would be guided by identity elements in an “operational code” on the acceptor stem [60]. Identity elements for tRNAs expanded in time to include the anticodon and bases in other parts of the tRNA upon their appearance: The major identity elements of *Bacillus subtilis* TrpRS for instance are located at discriminator G73 and the anticodon, and minor ones at A1-U72, G5-C68 and A9 [72,73]. According to evolutionary timelines, the recognition of anticodon as identity element by eARS did not take place until the age of the post-PTC ribosome [67].

The successive additions of different tRNA substructures raise the question of whether the final cloverleaf structure represents the result of chance or definable evolutionary factors. Interestingly, the cloverleaf structure is not confined to tRNA but occurs widely in biological systems, playing key roles in replication of RNA viruses of bacteria and plants, retroviral replication, *etc.*, which has led to the *genomic tag hypothesis* that the cloverleaf structure could confer a replication advantage prior to the advent of protein synthesis by facilitating the binding of replicase to the 3′ end of a genome, withdrawing a genomic RNA from the replicative pool by blocking the binding of the tag to replicase, or promoting RNA peptidation in the Peptidated RNA World. The hypothesis also postulates that the top half of tRNA consisting of acceptor stem and T arm is more ancient than the bottom half consisting of the anticodon and D arms [74].

The abundance of repetitive retroposons like MIR (mammalian-wide interspersed repeat) elements, which are found in all mammalian genomes as well as marsupials, with a tRNA cloverleaf-like “anticodon loop” that comprises six instead of seven base residues and accounting for 1%–2% of human DNA [75], is in accord with the enhancement of replication by tRNA-like sequences (TRLS). Another advantage of TRLS is their adaptability to functional recruitment exemplified by the exaptation of MIR sequences into exons and enhancers [76,77,78]. Direct evidence for replication enhancement by TRLS is furnished by suppressive mutants of Mauriceville and Varkud mitochondrial plasmids of *Neurospora* that contain a sequence insertion related to tRNA^Trp^, tRNA^Gly^ or tRNA^Val^, which gave rise to 25- to 100-fold overproduction of plasmid transcripts [79]. The involvements of TRLS in RNA viruses [74] also support the amplification and functional recruitment of TRLS in the RNA and Peptidated RNA Worlds. Moreover, because retroposons are mobilized via an RNA intermediate and integrated into chromosomes more or less randomly through retroposition, it has been suggested that the process might be a continuation of the ancient transition from RNA genome to DNA genome [80]. That retroposons are seldom if ever found in prokaryotes might be ascribable to the selective disadvantage of gene disruption caused by mobile element insertions in gene-dense prokaryotes rather than an exclusive retroposon-Eukarya relationship [81]. Since the adoption of tRNA as an adaptor in translation depended on a spectrum of varied TRLS to interact with specific ARSs, accommodate all the genetically encoded amino acids, and read all codons with high accuracy, the propensities of TRLS toward facile amplification and functional recruitment constituted important attributes. Thus, the assembly of different tRNA substructures into a cloverleaf was likely to be directed more by the inherent properties of TRLS than by chance.

An important advantage of the cloverleaf structure is that, because codon-anticodon binding occurs at a distance from the 3′ terminus, a change in the amino acid moiety at the 3′ terminus does not affect codon reading. Accordingly, pretran synthesis is allowed, producing Gln-tRNA from Glu-tRNA, Asn-tRNA from Asp-tRNA, Sec-tRNA from Ser-tRNA, and Cys-tRNA from *O*-phosphoSer(Sep)-tRNA in the course of genetic code expansion to include biosynthetically derived amino acids.

## 5. Origin of Genetic Code

Once tRNA evolution gave rise to triplet codon-anticodon pairing, deciding on the amino acids to admit into the code and allocating codons to them constituted the two foremost issues of code evolution. Based on the biosynthetic imprints in the code in the form of enriched contiguities (*viz.* being one base difference apart) between the codon domains of biosynthetically related amino acids, the coevolution theory of the genetic code (CET) proposes that, although numerous amino acids in the code were obtainable from prebiotic sources, some of the encoded amino acids originated from biosynthesis [82,83,84].

A large majority of the 20 encoded amino acids have been synthesized under prebiotic type conditions: Eleven of them including Met in fair yield via acrolein are obtainable from electric discharge, the concentrations of Phe and therefore Tyr via hydroxylation are expected to be substantial in the oceans through abiotic synthesis from phenylacetylene, Cys is prebiotically available from photochemical reaction and from dehydroalanine, Asn and Gln are produced from hydrolysis of nitriles prior to the formation of Asp and Glu, and there is also a reasonable abiotic synthesis of Trp from indole (in good yield from pyrolysis of hydrocarbons and ammonia) and dehydroalanine [85,86]. This leaves only Arg, His and Lys without evident prebiotic synthesis. However, as demonstrated by amino acids such as α-amino-n-butyric acid, norvaline and norleucine, which are obtainable from prebiotic sources but nonetheless excluded from the code, prebiotic availability is clearly not the sole determinant of encoding by the universal code. Instead, instability and biosynthetic factors also require consideration:

(i) Gln and Asn are highly sensitive to thermal hydrolysis: For this reason, the primordial oceans contained only ≤ 3.7 pM Gln and ≤ 24 nM Asn [87]. The more thermostable albizzine (α-amino-β-ureidopropionic acid) has thus been suggested as a possible early substitute for Gln [85].
(ii) The single codon assignments to Met and Trp strongly indicate that they are late arrivals supplied by biosynthesis.
(iii) The 20 encoded amino acids give rise to 190 pairs. The Cys-Trp pair ranks as the chemically most unlike pair, with the largest chemical distance of 215 compared to the minimum distance of 5 for the Leu-Ile pair, yet they are assigned codons in the same UGN box, which provides an unambiguous biosynthetic signal that the UGN codons are former Ser codons that have been apportioned to the Ser biosynthetic products Cys and Trp [88]. This biosynthetic signal is validated by the remarkable discoveries of allocation of part use of the UGA codon to selenoCys (Sec) via pretran synthesis of Sec-tRNA from Ser-tRNA [89], and the allocation of UGY codons to Cys via pretran synthesis of Cys-tRNA from Sep-tRNA [90].
(iv) Phe and Tyr as in the case of Trp and His are easily degraded by UV radiation: They were > 50% destroyed by irradiation for 48 h at pH 7 under an energy flux of 1.8 mW/cm^2^ [91]. However, whereas Gln and Asn could not hide from thermal degradation, prebiotically synthesized Phe and Tyr might find some shielding from UV radiation behind rocks or in ocean depths.

Based on the prebiotic synthesis, chemical instability and biosynthetic factors, CET postulated that the 20 encoded amino acids can be classified into the Phase 1-prebiotically sourced amino acids Gly, Ala, Ser, Asp, Glu, Val, Leu, Ile, Pro and Thr, and the Phase 2-biosynthetically sourced amino acids Phe, Tyr, Arg, His, Trp, Asn, Gln, Lys, Cys and Met [92] (Table 1). Among them, Pro and Thr were regarded as borderline Phase 1, and Phe and Tyr as borderline Phase 2. This classification is in substantial accord with irradiated synthesis employing high-energy particles [93,94], amino acid content of carbonaceous chondrite meteorites [12,95], and electric discharge synthesis [85,86]. Notably, all the lines of evidence in Table 1 point to the essentiality of both Phase 2 and Phase 1 amino acids as sources of the encoded amino acid ensemble. This dual sourcing of the encoded amino acids verifies not only the coevolution of genetic code and amino acid biosynthesis, but also a heterotrophic rather than autotrophic origin of life [8].

The number of alternate 64-codon genetic codes capable of allocating 1–6 codon packets to 20 amino acids is an astronomical 2 × 10^19^. As shown in Box 1, CET through subdivisions of the codon domains of Phase 1 amino acids to supply codons to Phase 2 amino acids reduces the number of potential alternate codes by a factor of 10^−11^ [96]. Error Minimization based on reduction of the perturbation occasioned by single base mutations and reading errors through the selection of codes where neighboring codons encode physicochemically similar amino acids reduces potential alternate codes by 10^−6^ [97]. Stereochemical interactions between amino acids and codons further reduces potential alternate codes by 4 × 10^−4^ [98]. Acting together, these three mechanisms yielded 4 × 10^−21^ selection, which sufficed to bring about the selection of one standard code out of 2 × 10^19^ alternatives.

**Figure 3:**
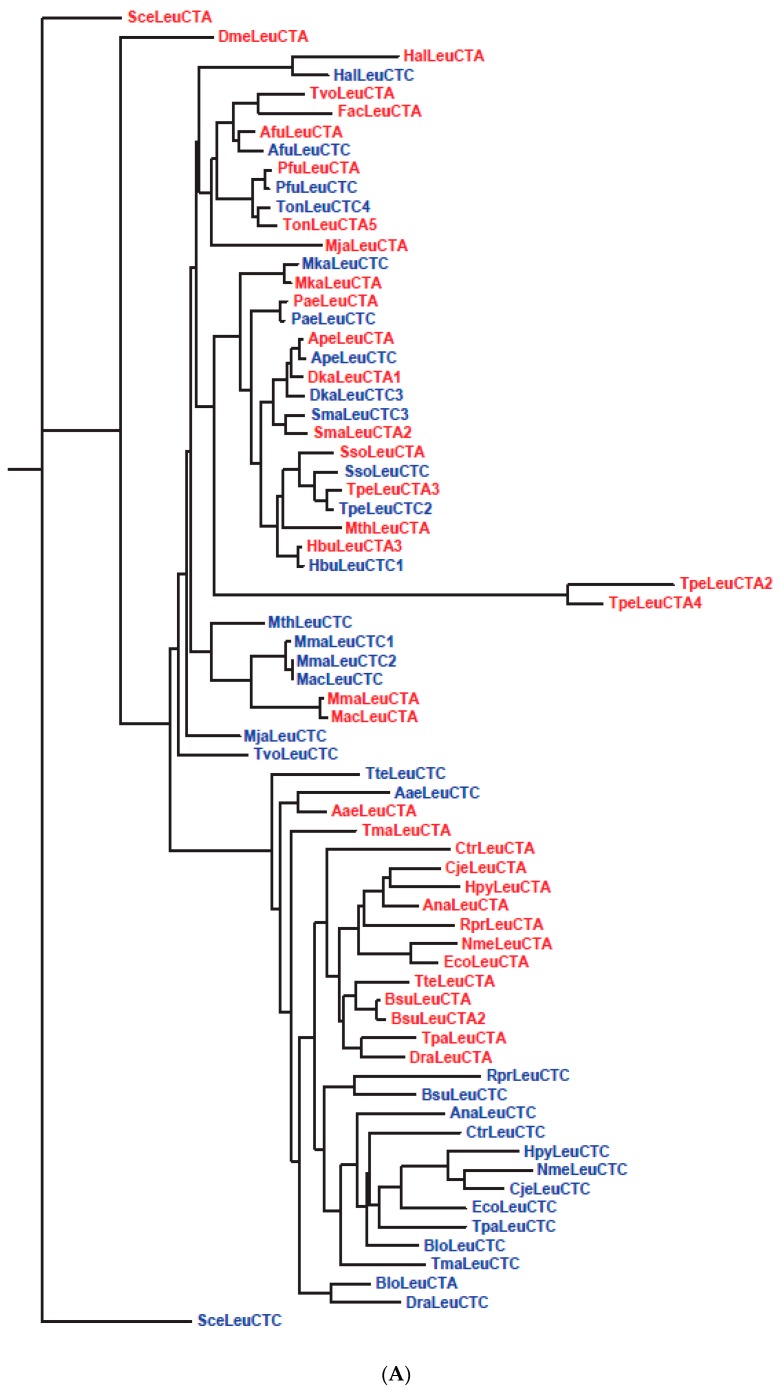

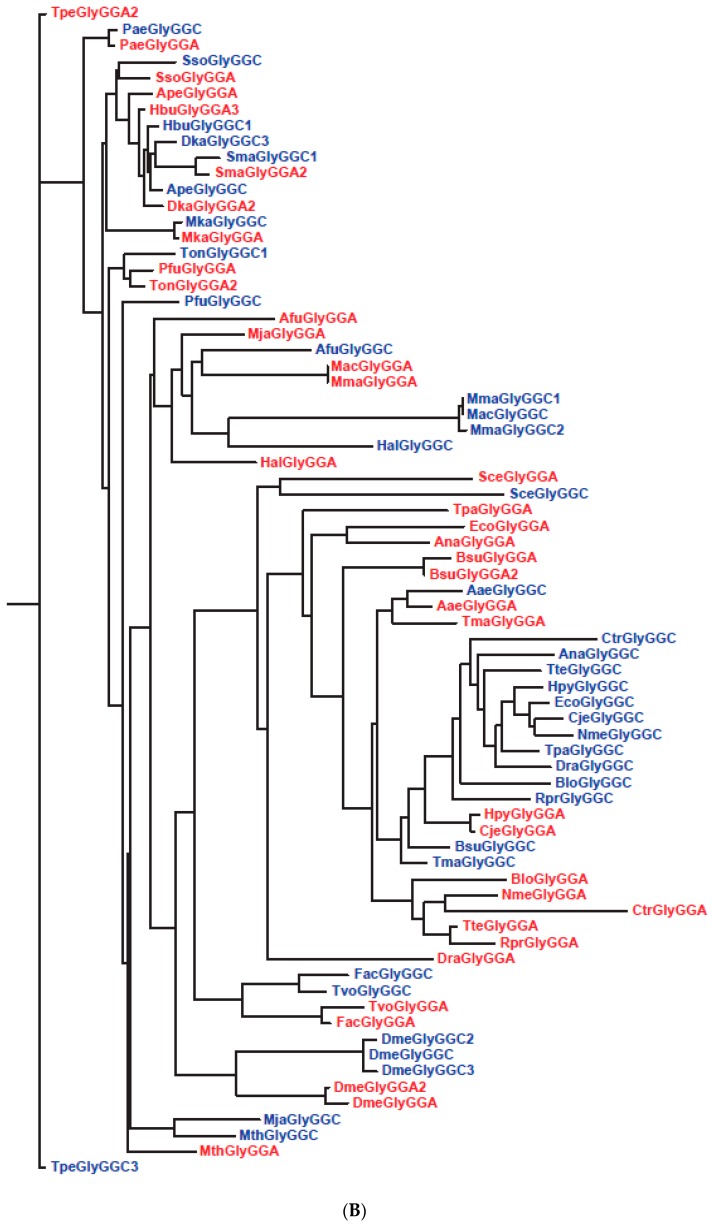

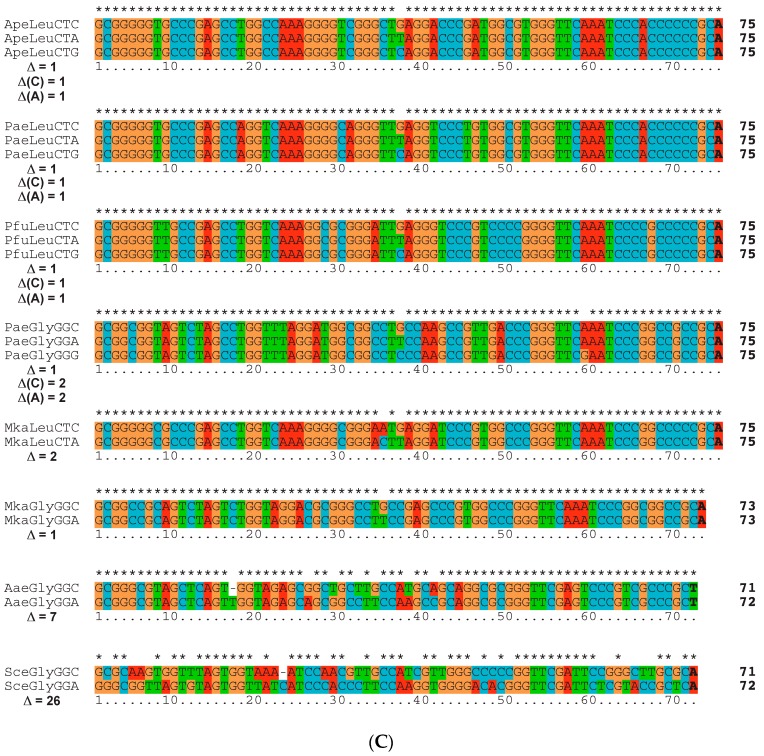
Transfer RNA genes for Leu and Gly codon boxes. (**A**) Gene tree for tRNAs complementary to CUC and CUA codons for Leu; and (**B**) gene tree for tRNAs complementary to GGC and GGA codons for Gly. Each tRNA gene branch is designated by three-letter species name, encoded amino acid and complementary codon (in blue and red for different codons); (**C**) Sequence alignments of examples of paired Leu or Gly tRNAs. Δ represents number of base differences between aligned NNC and NNA codon-complementary tRNAs, Δ (C) that between aligned NNG and NNC codon-complementary tRNAs, and Δ (A) that between aligned NNG and NNA codon-complementary tRNAs. Notably, all the closest pairs are archaeal ones. Three letter species names: **ARCHAEA. Crenarchaeota:** Ape *Aeropyrum pernix*, Dka *Desulfurococcus kamchatkensis*, Hbu *Hyperthermus butylicus*, Pae *Pyrobaculum aerophilum*, Sma *Staphylothermus marinus*, Sso *Sulfolobus solfataricus*, Ton *Thermococcus onnurineus*, Tpe *Thermofilum pendens*, **Euryarchaeota:** Afu *Archaeoglobus fulgidus*, Fac *Ferroplasma acidarmanus*, Hal *Halobacterium NRC-1*, Mac *Methanosarcina acetivorans*, Mja *Methanococcus jannaschii*, Mka *Methanopyrus kandleri*, Mma *Methanosarcina mazei*, Mth *Methanothermobacter thermautotrophicum*, Pfu *Pyrococcus furiosus*, Tvo *Thermoplasma volcanium*. **BACTERIA**. Aae *Aquifex aeolicus*, Ana *Anabaena* sp., Blo *Bifidobacterium longum*, Bsu *Bacillus subtilis*, Cje *Campylobacter jejuni*, Ctr *Chlamydia trachomatis*, Dra *Deinococcus radiodurans*, Eco *Escherichia coli*, Hpy *Helicobacter pylori*, Nme *Neisseria meningitidis*, Rpr *Rickettsia prowazekii*, Tma *Thermotoga maritima*, Tpa *Treponema pallidum*, Tte *Thermoanaerobacter tengcongensis*. **EUKARYA.** Dme *Drosophila melanogaster*, Sce *Saccharomyces cerevisiae*. Transfer RNA gene sequences were derived by Marck and Grosjean [105] or using tRNAscan [106], and gene trees built using the fitch distance method in PHYLIP [107].

**Figure 4:**
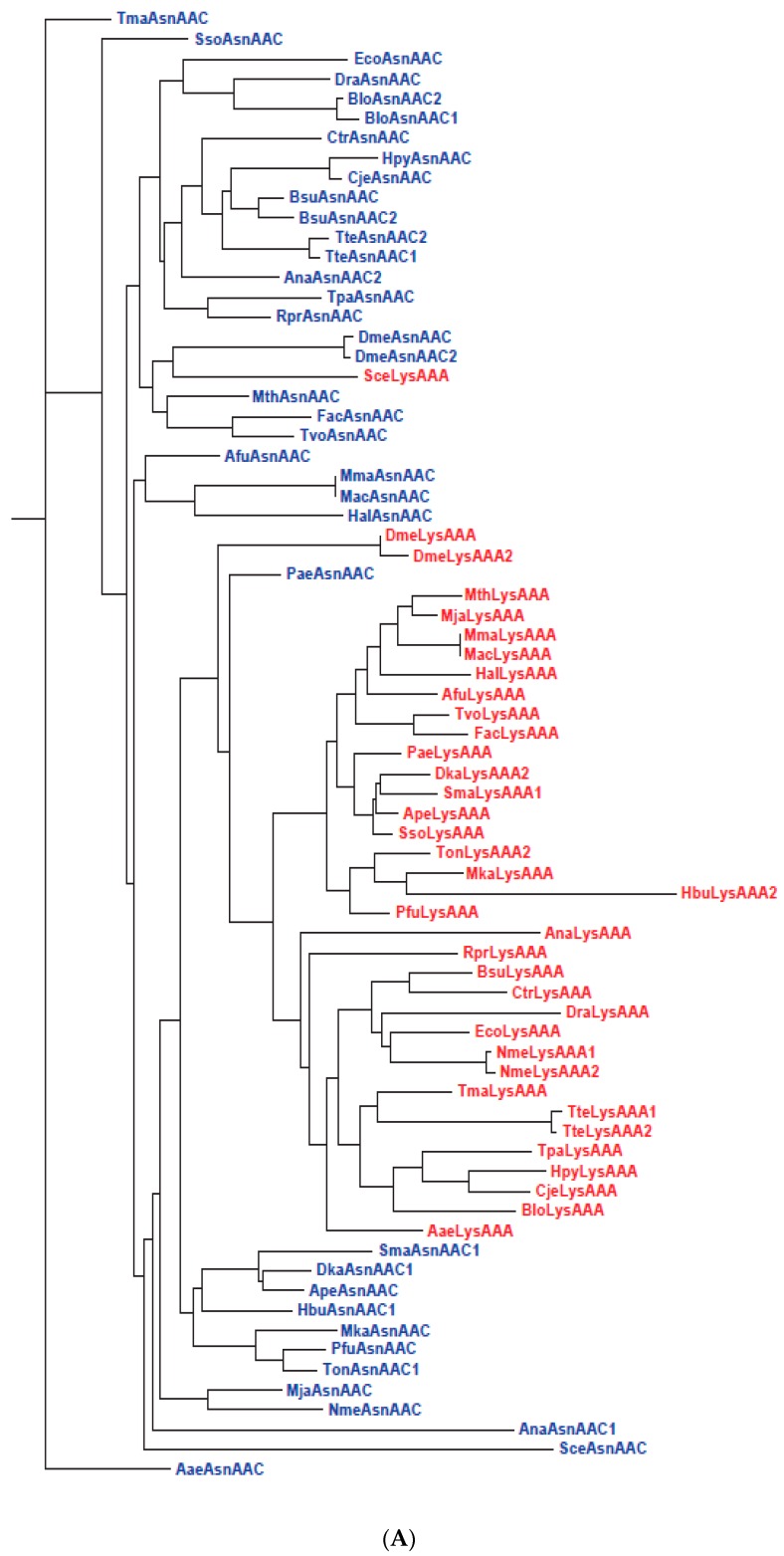

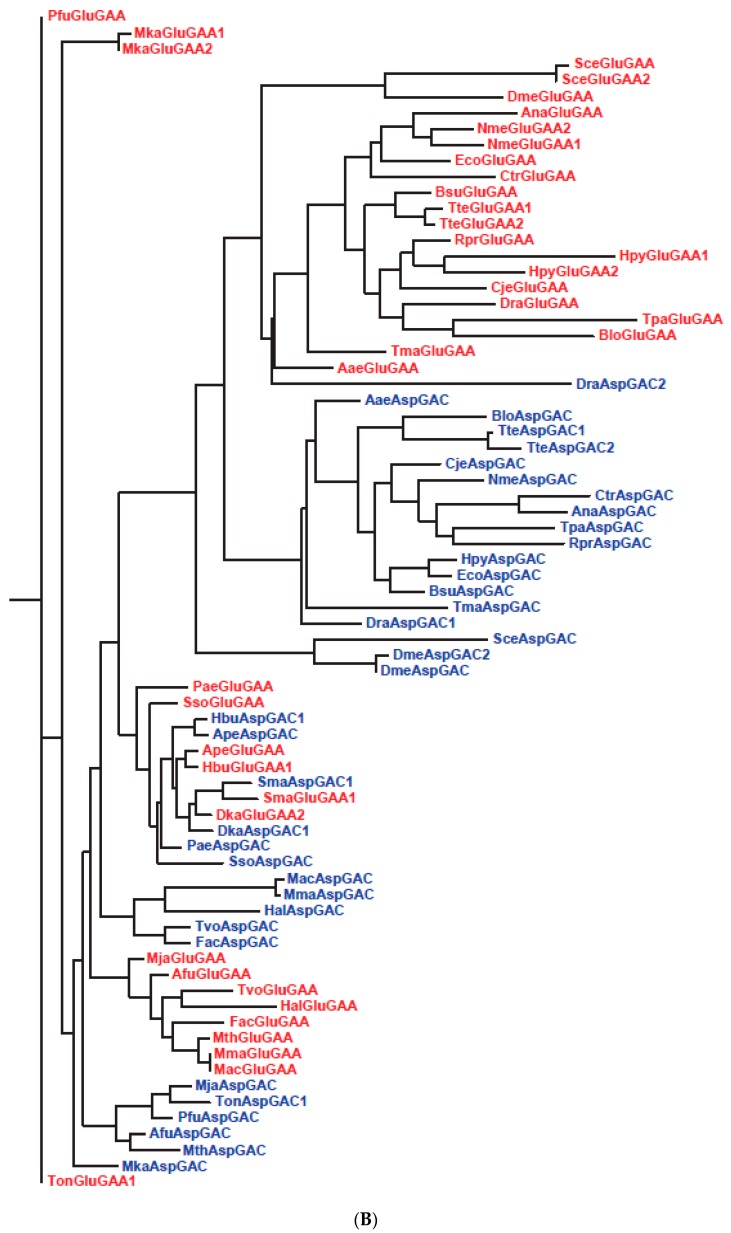

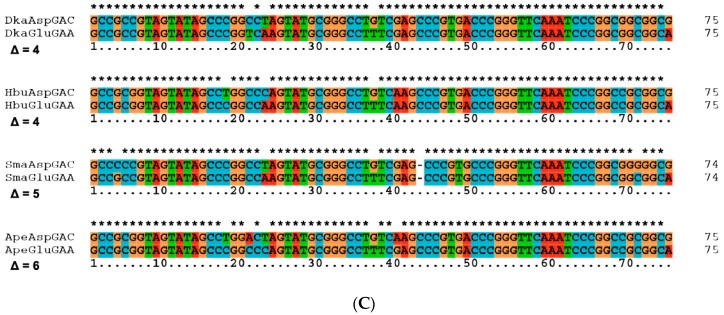
Transfer RNA genes for Asn/Lys and Asp/Glu codon boxes. (**A**) Gene tree for tRNAs complementary to AAC codon for Asn and AAA codon for Lys; and (**B**) gene tree for tRNAs complementary to GAC codon for Asp and GAA codon for Glu; (**C**) Sequence alignments of closely paired tRNA genes for Asp and Glu. Note: On the tree in part B, because of the only two-base difference between ApeAspGAC and HbuAspGAC, and likewise between ApeGluGAA and HbuGluGAA (Figure S2B), the Asp and Glu tRNA genes from either Ape or Hbu are not the most closely paired with one another but with its counterpart tRNA genes in the other species.

Interestingly, although the UNN, CNN and ANN codons are assigned to both Phase 1 and Phase 2 amino acids, GNN codons are assigned exclusively to Phase 1 amino acids, *viz.* Val, Ala, Asp, Glu and Gly. It is also puzzling why Asp and Glu, precursors to two large biosynthetic families of amino acids, retained between them the GAN codons while they gave away numerous erstwhile codons in other codon rows to amino acids that are their biosynthetic products. There are two possible explanations for these biases: The earliest codes began using only the GNN codons and no UNN, CNN or ANN codons, as in the proposals of RNY, GC, GNC and four-column types of primeval genetic codes [99,100,101,102,103,104], or the GNN codons functioned more proficiently in the early codes than UNN, CNN and ANN codons so that they were preferentially allocated to the abundant Val, Ala, Asp, Glu and Gly [8]. To assess the efficiency of GNN codons relative to other codons, the phylogentic trees for GNN and UNN anticodon-bearing tRNAs for standard 1aa and 2aa codon boxes (which give the four codons in a box to one amino acid or equally to two amino acids respectively) from different rows are examined in Figure 3 and Figure 4.

Figure 3A,B show the gene tree for the isoacceptor LeuCTC and LeuCTA tRNA (each named after the complementary codon for the tRNA) gene pair in the CUN codon box, and the isoacceptor GlyGGC and GlyGGA tRNA gene pair in the GGN codon box. Sequence alignments of some of the closely clustered pairs on these two trees are illustrated in Figure 3C, where the striking sequence conservation between the paired archaeal tRNA genes of ApeLeu, PaeLeu, PfuLeu, PaeGly and MkaGly, displaying in each instance only the single base difference (Δ = 1) in the anticodon, strongly points to the origin of each pair from an ancient gene duplication event. Furthermore, the ApeLeu, PaeLeu, PfuLeu and PaeGly codon boxes (but not the MkaLeu or MkaGly boxes) each contain a third LeuCTG or GlyGGG isoacceptor tRNA sequence, which is also included in the alignments in the figure. The base differences between this LeuCTG sequence and the LeuCTC and LeuCTA sequences are represented by Δ (C) and Δ (A) respectively, and the same applies to the base differences between the GlyGGG sequence and the GlyGGC and GlyGGA sequences. That Δ (C) and Δ (A) equal 1 or 2 for the ApeLeu, PaeLeu, PfuLeu and PaeGly codon boxes further indicates that the LeuCTG or GlyGGG tRNA sequences in these boxes are also related to the LeuCTC and LeuCTA, or GlyGGC and GlyGGA sequences respectively by ancient gene duplication events.

Box 1 **Reception-Seating Model of Alternate Codes [84,96]**

The elimination of vast numbers of alternate genetic codes by dividing the codons first among Phase 1 amino acids, followed by subdivision of the Phase 1 codon domains to include Phase 2 amino acids may be compared to the economized seating arrangements for guests at a wedding reception. There are 20 guests, A, B,^…^S,T, and twenty seats. There will be a total of p!(q!)^p^ different seating arrangements, where p is the number of seating sections, and q is the number of heads per section. In the first seating approach, all 20 guests are treated as a single group, drawing lots to determine seat assignments, so that p = 1 and q = 20. This yields a total of 2.4 × 10^18^ different seating arrangements for the guests.

Given there are five affinity groups of guests, four per group—(1) A–D are bride’s relatives; (2) E–H are groom’s relatives; (3) I–L are bride’s coworkers; (4) M–P are groom’s coworkers; and (5) Q–T are neighbors, a second seating approach is to divide the seats into five sections, and randomly draw lots to allocate these sections to groups 1–5. The four seats within each section are randomly distributed to the four individuals within the same group. In this case, p = 5 and q = 4. This yields a total of only 9.6 × 10^8^ seating arrangements. Thus, the subdivision constraint in the second approach disallowing mixed-group seatings within any section reduces the number of possible seating arrangements by a factor of 4 × 10^−10^.

Likewise, when the 64 codons in the genetic code are distributed to the 20 amino acids without any affinity grouping, the number of possible codes differing in codon allocations is approximately 2 × 10^19^. If the 20 amino acids are divided into biosynthetic affinity groups, the number of possible codes becomes 2 × 10^8^. This reduction of allowable alternate codes by a factor of 10^−11^ greatly facilitates the selection of a unique code out of all possible alternate codes.

In standard 1aa codon boxes such as the CUN and GGN boxes, the paired GNN and UNN anticodon-bearing tRNAs are isoacceptors for the same amino acid, and a high degree of sequence conservation between them incurs no misreading risk. In standard 2aa boxes, however, the paired tRNAs are alloacceptors for two different amino acids, and increased sequence separation between them might be important for reduction of cross-reading by one another’s cognate eARS. This is verified by the AsnAAC and LysAAA gene tree for the Asn/Lys codon box: Their same-species gene branches for Archaea, Bacteria and Eukarya species are well segregated into separate clusters on the tree (Figure 4A). Similar segregations of corresponding alloacceptor gene branches are likewise displayed by Archaea, Bacteria and Eukarya species on the trees for the 2aa Phe/Leu, His/Gln and Ser/Arg codon boxes (Figure S1). In contrast, although Figure 4B shows segregation of the AspGAC and GluGAA gene branches into separate clusters among Bacteria, Eukarya and some Archaea species, these two gene branches remain closely paired for the archaeons Dka, Hbu, Sma and Ape on the tree, with a base difference Δ of only 4–6 between the paired gene sequences (Figure 4C). Moreover, based on sequence alignments for the 22 archaeons, 34 bacteria and 7 eukaryotes, the average Δ-value for this tRNA pair for Asp and Glu is 13.2 for Archaea, 30.8 for Bacteria, and 24.3 for Eukarya (Figure S2), pointing to their closer pairing among Archaea relative to Bacteria and Eukarya.

The tardy segregation of GNN and UNN anticodon-bearing archaeal tRNAs in the GAN box for Asp/Glu compared to the AAN box for Asn/Lys, UUN box for Phe/Leu, CAN box for His/Gln and AGN box for Ser/Arg confirms that decoding by GNN and UNN anticodons in translation was more accurate in the GNN codon row than other rows in the primitive genetic codes, as suggested earlier [8]. This functional advantage of the GNN codons over other codons renders the RNY, GC, GNC and four-column types of primeval genetic codes groundless. Instead, it provides a functional rationale for the preferential allocation of the proficient GNN codons to the prebiotically abundant Val, Ala, Asp, Glu and Gly so that their usages in primordial translation could be maximized, and for the retention by Asp and Glu of their superior GAN codons while they gave away numerous codons in other rows to their biosynthetic products during coevolution of genetic code and amino acid biosynthesis.

## 6. Origin of Extant Life

The adoption of a near universal genetic code by all extant life suggests that the three living domains Bacteria, Archaea and Eukarya stemmed from the same root of life, or the last universal common ancestor (LUCA). Although the Bacteria domain was favored by early studies of protein paralogs as the likely host domain for LUCA [108], the number of protein paralogs analyzed was too few to be reliable, and the question was raised whether the root of life might ever be found [109]. However, the D_allo_ values, which measure the genetic distances between alloacceptor tRNAs that accept different amino acids in various species as index of tRNAome primitivity (Line 1, Table 2), indicate the greater primitivity of Archaea, especially the hyperthermophile methanogen archaeon *Methanopyrus kandleri* (Mka), than Bacteria and Eukarya [110]. The distinctly closer clustering of archaeal Leu or Gly gene pairs in Figure 3 compared to bacterial and eukaryotic gene pairs furnishes a graphic demonstration in this regard. Moreover, the conclusion of an Mka-proximal LUCA is supported by eARS distances and other evidence in the table. In the case of eARS, the parameter Q_ARS_ measuring the genetic similarity between eARS paralogs (Line 4) is highest for Mka at 138.5, far surpassing two other archaeons, *viz.*, Mth in second place at 119.3 and Mja in third place at 115.2 [111]. ValRS is also rooted in the Archaea by paralogous rooting (Line 5) through the analysis of sufficiently large numbers of ValRS and IleRS sequences [112]. The slower separation of the AspGAC and GluGAA tRNA genes in Archaea relative to Bacteria and Eukarya shown by the sequence alignments and average Δ-values in Figure 4C and Figure S2 adds evidence Line 26 to Table 2 for the primitivity of Archaea.

**Figure 5:**
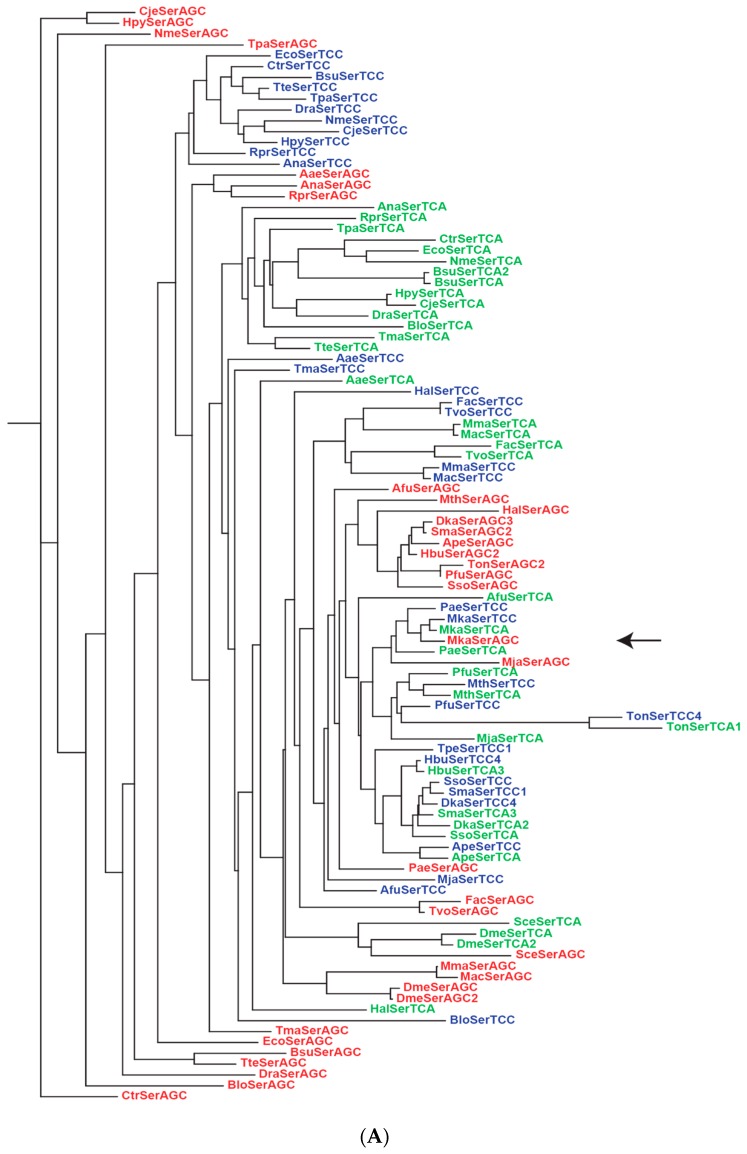

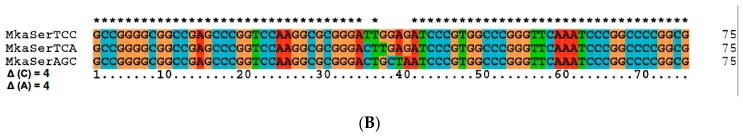
Transfer RNA genes for Ser from UCN and AGY codon domains. (**A**) Gene tree for the SerTCC (blue), SerTCA (green) and SerAGC (red) genes. Arrow points to MkaSerAGC gene branch; (**B**) Sequence alignments of three Ser tRNA gene sequences from *Methanopyrus kandleri*. Δ(C) represents number of base differences between SerAGC and SerTCC, and Δ(A) represents number of base differences between SerAGC and SerTCA.

It has been a long standing puzzle whether the apparently disconnected UCN and AGY codon domains of Ser arose from divergent or convergent evolution. In view of the extreme sequence conservation displayed by some archaeal tRNAs in the Leu and Gly codon boxes, the phylogenetic tree for Ser tRNAs from the UCN and AGY domains was examined to ascertain if any sequence conservation might remain detectible between the tRNAs from these two domains. As shown in Figure 5A, the same-species SerTCC (blue), SerTCA (green) and SerAGC (red) gene branches are largely placed into distinct clusters on the tree, although some mixing of blue and green archaeal branches is also detected. The three types of gene branches are, however, closely clustered together in the case of Mka, pointing to evident sequence conservation. Sequence alignment of these three tRNAs shows only a 4-base difference within the anticodon loop between SerAGC and either SerTCC or SerTCA, including a 2- or 3-base difference between the anticodon triplets (Figure 5B). This sequence conservation displayed by the Ser tRNAs of Mka is unmatched by any of the other 21 archaeal, 34 bacterial and 7 eukaryotic sequence trios aligned in Figure S3, where the archaeons Afu, Pae and *Pyrococcus horikoshii* (Pho) show a much higher, albeit next-lowest to Mka, 9-base difference between their SerAGC and either SerTCC or SerTCA genes. The preservation of a zero-base difference outside the anticodon loop between the three Mka Ser tRNAs from the UCN and AGY domains thus supplies an extraordinary missing link between the two codon domains, which establishes their common evolutionary origin, and contributes Line 27 to Table 2 for the unique primitivity of Mka.

Recent analysis of gene ontology (GO) terms shows that thermophilic and hyperthermophilic crenarchaeons such as Dka, Tpe, Sma, Hbu and Ton are among the oldest forms of life [136], contributing Line 28 to Table 2. The D_allo_ values estimated as described earlier are 0.455 for Dka, 0.576 for Tpe, 0.430 for Sma, 0.546 for Hbu and 0.600 for Ton. The D_allo_ values for Dka and Sma are lower than those for all Archaea, Bacteria and Eukarya species previously analyzed, except for the euryarchaeon Mka (0.351), and the crenarchaeons Ape (0.402) and Pae (0.408), in keeping with the placement of a LUCA near the junction between Euryarchaeota and Crenarchaeota [110].

## 7. Origins of Intron and Triplet Codon

Introns occur in pre-mRNA, tRNA and rRNAs, and their splicing mechanisms vary between biological domains. Pre-mRNA introns are abundant in Eukarya but confined among Archaea to homologs of eukaryotic Cbf5b (centromere binding factor 5) of some crenarchaeons. While rRNA introns are relatively few in number, tRNA introns are found in all three domains including all archaeal species [139,140,141,142,143,144]. There has been a long-running debate between the introns-early and introns-late views with respect to eukaryotic spliceosomal pre-mRNA introns [145,146], and it is suggested that introns-early applies only to about 30%–40% of present-day pre-mRNA introns *viz.* the earliest phase-0 introns [147].

Regarding the relative ages of pre-mRNA, tRNA and rRNA introns, it has been suggested that self-splicing bacterial tRNA introns are much older and ancestral to protein-spliced introns of archaeal and eukaryotic tRNA genes and nuclear spliceosomal introns [148]. However, any question relating to the first-appearance domain is unanswerable as long as the relative primitivities of Bacteria, Archaea and Eukarya remain undetermined. The overwhelming evidence for the greater primitivity of Archaea, especially LUCA-proximal *Methanopyrus* compared to both Bacteria and Eukarya (Table 2), establishes that archaeal tRNA introns are the oldest among cellular introns. The greater primitivity of archaeal tRNA introns compared to their pre-mRNA and rRNA introns is supported by the finding of endonuclease-spliced pre-mRNA or rRNA introns so far only in crenarchaea, not in euryarchaea [149,150], and the greater propensity of crenarchaea than euryarchaea either to employ more relaxed bulge-helix-bulge (BHB) motifs as splicing guides or to place some of the tRNA introns outside the anticodon loop [151,152,153], which suggests that, of the three types of archaeal introns, only the tRNA introns were prominent prior to the deep Crenarchaeota/Euryarchaeota divide [115]. The similarity between the splicing endonucleases employed by Archaea to splice their tRNA introns and the hammerhead ribozymes in the generation of 5′-OH and 2′,3′-cyclic phosphate termini further suggests that the splicing endonuclease mechanism could be derived from a ribozyme-based reaction [142].

The evolutionary timeline for protein folds suggests that Archaea are older than Bacteria and Eukarya by eons [68,134]. Interestingly, the archaeal endonuclease splicing mechanism is adopted with limited change by eukaryotes for their tRNA introns: 75% archaeal and all eukaryotic tRNA introns are inserted at position 37/38 in the anticodon loop one base 3’ to the anticodon, and both archaeal and eukaryotic tRNA splicing endonucleases are guided by the BHB motif. Bacteria and bacteriophages, however, employ self-splicing Group I introns instead of endonuclease-spliced introns. Eukarya are eclectic in their approach to introns. They adopt endonuclease-spliced introns for their tRNAs and Group I introns for their rRNAs, whereas their pre-mRNA introns are spliced by a spliceosomal machinery in the nucleus that is postulated to be derived from Group II introns based on the formation of a lariat intermediate and similarities between U6/U2 RNAs and domain V of Group II introns [150].

The foremost antiquity of archaeal tRNA introns poses the question with respect to the nature of the evolutionary incentives that could have led to the innovation of introns. The potential advantages include the following.

### 7.1. Exon Shuffling

Introns can promote exon shuffling to produce novel genes in evolution, which is important to cell surface and extracellular proteins [145,154] and has been demonstrated with the self-splicing introns of bacteriophage T4 [155].

### 7.2. Exon Regulation

The presence of introns in a gene necessitates RNA splicing, and enables the regulation of gene expression through the modulation of splicing activity, the perturbation of which can be important to human disease etiologies [156,157].

### 7.3. Exon Diversification

Introns are known to give rise to novel exons through exonization and alternative splicing. Additionally, in primitive systems, intron splicing can be expected to be noisy and imprecise, producing exon sequence variations near the spliced junction. Comparable sequence imprecisions have found important applications even today. Because most transposons are excised imprecisely, leaving behind small DNA variations or footprints at the sites of excision, they add to the DNA sequence diversity needed for evolution through alterations in amino acid sequences caused by their excision [158]. As well, the removal of intervening sequence followed by the joining of antibody gene segments is deliberately imprecise, causing the loss or gain of nucleotides to result in “junctional diversification” that amplifies the diversity of V-region sequences, especially in the third hypervariable region [159]. Notably, for pre-mRNA introns, splicing imprecision generates only variations in protein sequences. On the other hand, for tRNA and rRNA introns in the Peptidated RNA World, replication and/or recombination of imprecisely spliced intermediates and products could generate variations in the tRNA and rRNA genes.

In LUCA-proximal Mka, the introns found in eight tRNA genes are modest in length containing an average of only 40 nt [153]. They are therefore limited in effectiveness for exon shuffling. Moreover, because the intra-anticodon loop introns divide the tRNA into two halves breaking up both the acceptor stem and the anticodon stem, shuffling the two halves between different tRNA genes cannot produce an appropriately hydrogen-bonded acceptor stem or anticodon stem in the recombinant tRNA molecule, rendering the latter unstable and non-functional. Additionally, since primitive Mka displays relatively simplistic regulatory mechanisms [114], any contribution made by tRNA introns toward regulation of tRNA expression in LUCA’s ancestors would be limited in significance.

Accordingly, the major contribution made by the tRNA introns to pre-Lucans was likely to be anticodon loop diversification resulting from imprecise splicing of the primitive tRNA intron in the anticodon loop. The imprecision gave rise to mutations that facilitated the following:

(a) Expansion of the anticodon repertoire of the genetic code at different stages of tRNA evolution so that ample anticodons were made available to the evolving tRNAs as the tRNAome underwent expansion with recruitment of new anticodons.
(b) Continual variation of the loop sequence with concomitant variation in loop nucleoside modifications facilitated the fulfillment of wobble base pairing requirements. Notably, the estimated LUCA genome contained a number of nucleoside modifying enzymes [160], and the tRNAs of LUCA-proximal *Methanopyrus* is enriched with modified nucleosides including ac^6^A, which represents a “minimalist” nucleoside modification where the amino acid moiety in t^6^A is replaced by an acetyl function. The discovery of ac^6^A and two minimalist wyeosine-family nucleosides from Archaea suggests that tRNA nucleoside modifications are simpler in Archaea than in Bacteria or Eukarya [137,138], thus contributing evidence Line 29 to Table 2 in support of the primitivity of Archaea.
(c) Progressive enhancement of the codon-anticodon association constant on account of optimizations in anticodon loop sequence and nucleoside modifications, thereby enabling a reduction in anticodon size and complexity down to three bases to establish the triplet codons and anticodons of the modern genetic code (Figure 2).

The anticodon loop of tRNA, like the genetic code it underwrites, is a product of intense evolutionary engineering. Every anticodon loop in a tRNAome must accomplish a two-fold task: triplet codon-anticodon pairing with greatly enhanced association constants, and precise pairing of the anticodon with one or more codons in accordance with wobble geometries that may depart from the standard A-U and G-C complementary pairings. As a result, both the base sequence and the nature of modified nucleosides in the anticodon loop need to be highly optimized in order to implement wobble. Conversely, the stringent challenge faced by the evolving anticodon loops furnished an important incentive for both the innovation of intron and the insertion of the first introns into position 37/38 of tRNA, where anticodon loop diversification could be effective.

## 8. Origin of Wobble

The 64 codons in the genetic code are divided into 16 four-codon boxes. Wobble base pairings enable the reading of the four codons in any box by using less than four tRNA anticodons [161]. While bacterial and eukaryotic species typically utilize non-uniform combinations of anticodons to read different codon boxes, most archaeal species employ a highly uniform anticodon usage, *viz.* using a GNN–UNN–CNN anticodon-trio in the eight standard 1aa-codon boxes as well as the five standard 2aa-codon boxes [113,162]. Among the archaeal exceptions, a few species such as Mja, Mth and Tvo employ the GNN–UNN–CNN anticodon-trio along with the GNN–UNN anticodon-duo to read their standard boxes, and Mka employs uniformly the GNN–UNN anticodon-duo to read all its thirteen standard boxes [113]. The ultra-simplicity of the Mka uniform anticodon-duo usage provides evidence Line 3 in Table 2 in support of the proximity of Mka to a LUCA. Taken altogether, the evidence in the table point to Mka as LUCA-proximal, and hence its wobble mode as the most primitive wobble mode among extant organisms.

The surprising ultra-conservation displayed by some archaeons with a single base difference at the anticodon separating their GNN-, UNN- and CNN-anticodon bearing tRNAs within the same codon box (Figure 3C) indicates that these three tRNAs have descended from gene duplication events giving rise to two rounds of sequence divergence, which verifies the *cluster-dispersion model* of tRNA evolution where tRNA sequences underwent dispersion from a localized GC-rich sequence space region to expand in number through gene divergence and encode novel amino acids [110]. In the first round of divergence, the Mka usage of a GNN- and UNN-anticodon bearing tRNA pair to decode each of its standard codon boxes arose from an even more primitive stage of the genetic code when all four codons in some or all of the standard boxes were read by only a single tRNA, in accord with the findings that a single UNN anticodon is employed by a number of genetic codes to read more than one of their standard 1-aa boxes [113,162], and the engineering of a single UNN “superwobble” to read all four Gly codons [163]. Thus, a single UNN could serve as a Stage I superwobble anticodon to read all four codons in a codon box in the earliest codes, thereby limiting each codon box to encode only a single amino acid. Later on, with the divergence of the single UNN anticodon-bearing tRNA in a box into UNN- and GNN-anticodon bearing tRNAs, each box gained the potential to encode more than one amino acid. Otherwise the protein alphabet based on triplet codons could be limited to a maximum of 16 instead of 20 + 2 encoded amino acids. The second round of divergence produced a CNN-anticodon bearing tRNA.

Table 3 summarizes the different stages of wobble evolution. Starting with Stage I wobble with the usage of a single UNN-anticodon, the addition of a GNN-anticodon through tRNA gene divergence gave rise to the Stage II wobble of Mka based on the GNN–UNN anticodon-duo. The further addition of a CNN-anticodon again through tRNA gene divergence enabled the mixed usages of Stage II and Stage III wobbles by for example Mja, Mth and Tvo, as well as the usage by a majority of Archaea species of Stage III wobble to decode all their standard codon boxes. Thus, the transitions from Stage I to Stage II and hence to Stage III wobble have been achieved with the addition of one anticodon via gene divergence at each transition. Among the Archaea, so far only Fac carries an AAG anticodon on the FacLeuCTT gene [113].

The tempo of adding one anticodon per stage has continued in the transition of Stage III wobble to the Stage IV wobble employed by Bacteria and Eukarya based on the four-anticodon ensemble of GNN–UNN–CNN–A(I)NN. While some bacteria such as Tma, Bbu, Tpa, Mpn, Cje and Hpy do not employ any A(I)NN anticodon, other bacteria typically employ at least one A(I)NN anticodon and also non-uniform combinations of anticodons for their standard codon boxes. Eukarya on the other hand employ non-uniform combinations with frequent usage of an A(I)NN–UNN–CNN anticodon-trio for their 1aa boxes and a GNN–UNN–CNN anticodon-trio for their 2aa boxes [113]. Stage V wobble is based on the usage of a single UNN anticodon in a 1aa box in bacteria and organelles. It is striking that, although Stage V wobble is likely derived from a simplification of Stage II-IV wobbles, its usage of a single UNN-anticodon resembles Stage I wobble, bringing wobble evolution full circle back to its very beginning.

## 9. Origins of Biological Domains

The division of species of organisms into the three domains of Bacteria, Archaea and Eukarya represents an outstanding discovery in Darwinian evolution [172], and its causes need to be deciphered. Although the genomes from these different domains are readily distinguished from each other based on their characteristic rRNA sequences, the possible roles played by inter-domain variations in rRNA sequences, or the divergence between formylated and non-formylated Met-tRNAi, in bringing about domain separations are unclear. Some phenotypic differences between the domains, however, could contribute significant evolutionary incentives to bring about domain separations:

### 9.1. Anticodon Strategy

A key demarcation of Archaea from Bacteria and Eukarya resides in the conservatism of most archaeons in their narrow compliance to the Stages II-III wobble modes (Table 3), in contrast to the common adoption by Bacteria and Eukarya of Stages IV wobble coupled with non-uniform anticodon usages in different standard codon boxes [113]. This difference might be traceable to LepA (or EF4), which is one of the most highly conserved proteins, present in all bacteria and nearly all eukaryotes but not in Archaea. LepA enables back translocation during translation to prevent ribosome stalling, thereby enhancing ribosomal tolerance to changes in ionic concentrations [173]. Thus, bacterial and eukaryotic cells equipped with LepA could be more tolerant of internal milieu variations and therefore more adaptive to changing environments compared to archaeons. To cope with internal milieu variations, however, codon-anticodon pairing strengths might have to be fine-tuned for individual codons, leading to the varied anticodon combinations employed by Bacteria and Eukarya for different codon boxes in contrast to the much more uniform anticodon combinations employed by Archaea [174]. The parting of the bacterial and eukaryotic lineages from the archaeal lineage may therefore be viewed as a divergence between adventurist and conservative strategies in anticodon usage.

### 9.2. Membrane Lipids

Another factor distinguishing Archaea from Bacteria and Eukarya is the adoption of glycerol ether lipids by Archaea for membranes but glycerol ester lipids by Bacteria and Eukarya. Although numerous present-day archaeal and bacterial species might conceivably survive a switch from ether lipids to ester lipids or *vice versa*, under the historical Hot Cross Scenario [160,175], pre-Lucans facing dwindling supplies of prebiotic nutrients were drawn to the organics-rich surroundings of hydrothermal vents where a *Methanopyrus*-like LUCA in possession of the biochemical weaponry of a DNA genome and a 20aa genetic code arose; at these vents, the greater thermal stability of ether lipids relative to ester lipids toward hydrolysis was crucial to LUCA’s survival and success. However, when LUCA’s descendants subsequently spread from the hydrothermal vents back to mesophilic zones, eliminating all competing species to bring about the present-day genetic code and universal adoption of DNA genes, they encountered temperatures not as extreme as the ~110 °C growing temperature for *Methanopyrus* and LUCA. Thereupon, the adherence to ether lipids became non-essential, allowing a changeover to ester lipids by the last common bacterial ancestor and its offsprings. Eukarya, which might have inherited genes from both a *Ferroplasma*-like archaeon and a *Rickettsia*-like bacterium [112], also chose ester-lipids.

### 9.3. Nuclear Membrane

For Eukarya, the defining characteristic as expressed in their domain name is the possession of a nuclear membrane. Typically prokaryotic genomes contain up to ~50 M nucleotides, whereas eukaryotic genomes contain upwards from ~50 M nucleotides, which suggests that the enclosure of a nucleus by nuclear membrane enables a larger genome and extra genes [176]. Moreover, in prokaryotes, because transcription and splicing take place in the same compartment as translation, binding of ribosome to a growing pre-mRNA strand can interfere with pre-mRNA splicing or risk translation of an incompletely spliced pre-mRNA [140,148]. As a result, pre-mRNA introns are found only rarely in prokaryotic cells [141]. On the other hand, Eukarya with their nuclear membranes are free of this difficulty, and can engage in massive development of pre-mRNA introns with alternative splicing to enhance protein information content. A threshold genome/proteome size may also be prerequisite to multicellularity [8]. The nuclear membrane, by facilitating both increased genome size and proteome expansion through alternative splicing, therefore could offer Eukarya entry into otherwise unattainable lifestyles, and in so doing steer them away from Archaea and Bacteria to form their own distinct domain.

## 10. Origin of Synthetic Life

The coevolution theory of the genetic code, in postulating that the code expanded to accommodate the entry of biosynthetic Phase 2 amino acids into the code, contradicts the frozen accident theory of the code [177], for the encoding of Phase 2 amino acids was not accidental but driven by the side chain imperative. To test the CET prediction of a mutable code, experiments were performed on the deletion-based Trp-auxotrophic QB928 strain of *Bacillus subtilis* to determine whether the encoded Trp as a canonical amino acid (CAA) in the protein alphabet could be replaced or displaced by the noncanonical amino acid (NCAA) 4FTrp. The LC33 and LC88 mutants of QB928 showed that Trp can be replaced throughout the proteome by 4FTrp, 5FTrp and 6FTrp, all of which are normally highly toxic to the cells. Moreover, in the HR15 and HR23 mutants, Trp is not only replaced but actually displaced by 4-fluoroTrp, such that Trp loses its immemorial role as an essential protein building block. In the TR7-1, TR7-2 and TR7-3 reverants of HR23, the code is further mutated to restore to Trp its capacity as a competent building block [178,179,180].

Various NCAAs are known to compete against CAAs for attachment to tRNA and gain entry into cellular proteins. In favorable cases, some NCAAs can replace CAAs extensively in proteins, as illustrated by the isomorphous replacement of Met by selenoMet in growth of *E. coli* in suspension although not on agar in the presence of only selenoMet [181], replacement of all but 1.3% and 2.0% Met in T4 and *E. coli* thioredoxins [182], and replacement of all but 5.8% Met in *Pseudomonas* azurin [183]. However, such unmutated replacements of CAA by NCAA cannot reveal whether the code is by its nature mutable or immutable. In contrast, in the isolations of the LC33, LC88, HR15, HR23 and TR7 mutants, the mutability of the code was established for three different types of mutations: First, the code was mutated to render 4FTrp non-toxic and supportive of propagation for LC33, and similarly 4FTrp, 5FTrp and 6FTrp for LC88; secondly, Trp was rejected from the ranks of propagation-supportive amino acids and turned into a toxic inhibitor against propagation of HR23 on 4FTrp; and, thirdly, HR23 was mutated to TR7-1, TR7-2 and TR7-3 reinstating the ability of Trp to support cell propagation. Since the protein alphabet founded on the genetic code is the most basic attribute of living systems, it is suggested that a mutation as fundamental as the displacement of Trp by 4FTrp would yield an entirely new type of life [184]. Accordingly, cells that have mutated to the usage of an altered protein or nucleic acid alphabet can be designated as *synthetic life* to distinguish them from synthetic biology constructs that bear novel genomes but abide by the universal protein and nucleic acid alphabets [185]. On this basis, altered protein alphabets can result in either *optional* synthetic life forms where a CAA can be optionally replaced by an NCAA, or *mandatory* synthetic life forms where only the NCAA but not the original CAA can support life.

Synthetic life forms are steadily expanding: genome-wide replacements of Trp by 4FTrp and 6FTrp have been achieved with *E. coli* B7-3 and bacteriophage [186,187,188], and replacement of Trp by l-β-(thieno[3,2-*b*]pyrrolyl)alanine ([3,2]Tpa) in *E. coli* MT20 [189]. Moreoever, based on the finding that numerous eARS are domain-specific and display strikingly low reactivities toward tRNA from the other side of a schism between the Bacteria and Archaea-Eukarya blocs [190], the use of orthogonal eARS-tRNA pairs, e.g., archaeal TyrRS-tRNATyr from Mja, LeuRS-tRNALeu from Mth or GluRS from Pho with a consensal archaeal tRNAGlu has enabled the incorporation of NCAA into specific sites of the proteome in both prokaryotes and eukaryotes [191,192,193,194,195,196,197,198,199,200,201,202,203,204,205,206,207,208,209,210], as has been attained through phenotypic suppression [211]. The DNA alphabet has been altered as well, where the canonical deoxyribonucleotide (CDN) thymine is replaced by the noncanonical deoxyribonucleotide (NCDN) 5-chlorouracil, yielding both optional and mandatory synthetic life forms [212,213,214] (Table 4). Useful molecular insights enabled by the synthetic life forms are exemplified by the following.

The mutability of the genetic code and protein alphabet, with the rejection of Trp by HR15 and HR23 and its reacceptance by TR7-1, TR7-2 and TR7-3, suggests that the choice of amino acids by the genetic code is the result of intense competition and selection. This resolves the puzzle of why such prebiotically available amino acids as α-amino-*n*-butyric acid, norvaline and norleucine are absent from the code [85]: These amino acids either never made the cut against competitors like Ala, Val, Leu and Ile on account of lesser availability, or they were admitted into the code at one time but came to be rejected for inadequate performance relative to their competitors. Likewise, the A, G, T, U and C bases along with the ribose and deoxyribose constituents of nucleic acids were in all likelihood the end results of rigorous selection rather than accidental adoption by the living system. The same pertains to other universal cellular constituents such as glycerol lipids, ATP and NAD. In fact, the number of mutations converting *B. subtilis* wild-type QB928 into the synthetic life form LC33, LC33 into HR23, and HR23 into TR7-1, TR7-2 or TR7-3 are found by their genomic sequences to be relatively limited in number [215]. This reveals that an important factor for the three-billion-year stability of the protein alphabet consists of “oligogenic barriers” that comprise a small number of ultra-sensitive genes, and hence a small number of essential biosynthetic/metabolic pathways, which become dysfunctional upon the replacement or displacement of a CAA by an NCAA [180,215].

The utilization of NCAA brings unforeseen evolutionary pressure on proteins, and in so doing enables the investigation of otherwise difficult to explore protein sequence spaces. When the genomic sequence of the mandatory 4FTrp utilizer HR23 strain was compared to those of its Trp-utilizing TR7 revertants, the latter were found to harbor the β-Glu433Lys, β′-Ile280Thr or β′-Pro277His mutation in their RNA polymerase (RNAP). None of these three mutations, located on the two sides of the conserved outer claw-like region of RNAP, is found in any of 960 bacterial RpoB and 844 bacterial RpoC sequences. The uniqueness of these mutations suggests that HR23 in adaptation to the NCAA 4FTrp has brought its RNAP into an unusual protein configuration, such that rare mutations were required to readapt RNAP to the presence of Trp, thereby revealing hitherto hidden RNAP sequence spaces and configurations that are compatible with enzyme activity. Analysis of such hidden sequence spaces and configurations opened up by the incorporation of NCAAs will substantially widen the amplitudes of protein chemistry [215].

In biological evolution, the fitness of a species may be estimated in terms of survival and competition between species. In molecular evolution, fitness is more difficult to assess. However, in the case of bacteriophage T7, it was found that expanding the genetic code to admit the NCAA 3-iodoTyr into the type II holin protein resulted in higher fitness of the 3-iodoTyr39 bearing phage in competition against the Tyr39 bearing phage [216]. This experimental system not only demonstrates the actual attainment of improved biological fitness through beneficial expansion of the protein alphabet, but also shows how the fitness of alternate genetic codes and DNA base pairs can be evaluated in synthetic life forms.

Since synthetic life forms employing altered protein and DNA alphabets are novel bioentities, their investigation and application require stringent measures to ensure safety. In this regard, the novel protein or DNA alphabets of the mandatory synthetic life forms furnish built-in devices for biocontainment that render their proliferation controllable by the supply of an unnatural amino acid or deoxyribonucleotide. By inserting an essential NCAA into multiple genes, escape frequency of ~10^−11^ has been achieved, thereby providing effective biocontainment not only against the spread of the mandatory novel alphabets themselves, but also the spread of genetically modified organisms (GMO) that contain highly toxic genes [209,210].

## 11. Discussion

The emergence of life spans eight stages of development [8]:

Stage 1. Prebiotic synthesis
Stage 2. Functional RNA selection by metabolite
Stage 3. RNA World
Stage 4. Peptidated RNA World
Stage 5. Coevolution of genetic code and amino acid biosynthesis
Stage 6. Last universal common ancestor
Stage 7. Darwinian evolution
Stage 8. Synthetic life

The coevolution theory of the genetic code was proposed in 1975 to explain the origin of the structure of the genetic code [82]. Over the past four decades, genomics and allied sciences have turned the genomes of organisms into open books, greatly advancing our understanding of both biological and prebiotic evolution. The new data have brought verification of the basic tenets of CET, *viz.* biosynthesis contributed amino acids along with prebiotic synthesis to the code, pretran synthesis played a key role in conveying codons to some of the biosynthetically derived amino acids, thereby leaving indelible biosynthetic imprints on codon allocations, and the code is a mutable code open to alteration [8,83,217]. The pretran syntheses of Sec-tRNA from Ser-tRNA [89], Cys-tRNA from Sep-tRNA [90], and Asn-tRNA from Asp-tRNA [218,219,220] have added to the known pretran syntheses of Gln-tRNA from Glu-tRNA and fMet-tRNA from Met-tRNA to verify the pivotal role of pretran synthetic pathways in expanding the genetic code admitting biosynthetically derived Phase 2 amino acids, as well as the postulate of CET that the UGN codon box was part of the Phase 1 Ser codon domain. The confinement of the pretran synthetic SepRS pathway to the primitive methanogenic euryarchaeons Mka, Mth and Mja in contrast to the widespread horizontal gene transfer-assisted distribution of the direct CysRS pathway over all three living domains [90,221] is in accord with the antiquity of Mka, Mth, Mja (Line 8, Table 2) and the SepRS pathway.

Evolutionary timeline analysis finds that late development of tRNA and ribosomal functions such as anticodon loop recognition by eARS, and decoding and ribosomal protein synthesis with a mature PTC coincided with the development of amino acid biosynthetic pathways [67,222], pointing to a much later development of the expanded Phase 2 genetic code at the advent of Protein World compared to the initial Phase 1 code that guided peptide prosthetic group formation in the Peptidated RNA World. The fact that Trp and Met secured only one codon each, and Sec and Pyl just part of a codon each, suggests that Phase 2 code expansion came to an abrupt halt as soon as ribosomal protein synthesis succeeded in reducing the error rate of translation (see below), following which post-translational modifications took over the task of introducing novel amino acid side chains into proteins.

The coevolution of genetic code and amino acid biosynthesis not only brings about a 20 amino acid code but also reveals important evolutionary factors that are important to both the coevolution process itself and the understanding of events in other stages of life’s emergence.

### 11.1. Amino Acid-RNA Cooperation

The transition from Stage 1 to Stage 2 of life’s emergence was hindered by two pitfalls. First, abiotic RNA polymerizations gave rise almost exclusively to random non-functional sequences [5]. Secondly, template-directed RNA replication yielded dead-end double-stranded duplexes that were difficult to pull apart to renew replication [6,7,8]. The living state being a partnership of nucleic acids, proteins and lipid membranes, it may be expected that amino acid-RNA cooperation was instrumental to the establishment of this partnership. Genetic code expansion illustrates how conjugates between amino acids and tRNAs underwent pretran synthesis to expand the code, and it merits exploration whether amino acids/metabolites might likewise cooperate with RNA to overcome the difficulty of the Stage 1–Stage 2 transition. In this regard, analysis of metabolite-fRNA-template equilibria shows that metabolites could split up dead-end template-fRNA duplexes to activate REIM selectively for fRNA replication and launch Stage 2. Stage 2 in turn supplied the accumulation of fRNAs required for the realization of the RNA World at Stage 3.

### 11.2. Side Chain Imperative

CET indicates that the key incentive in code expansion adding Phase 2 amino acids to the code was the incessant demand by the side chain imperative for more side chains to improve protein performance, which remains manifest today in the large array of post-translational modifications fabricated by organisms. In the RNA World, this imperative drove the development of post-replication modifications that included the covalent attachment of amino acids and peptides to fRNAs, resulting in the growth and nurture of protein folds and domains in the polypeptide prosthetic groups on fRNAs to pave the way for the transition from Stage 3 to Stage 4. It is proposed that the Peptidated RNA World began with the appearance of protein folds and domains, and ended when the Protein World took over upon the advent of the post-PTC ribosome [8]. On this basis, since the biosphere started at ~3.8 Gya [223], the RNA World according to the evolutionary timelines [67] spanned ~0.2 Gy (3.8–3.6 Gya), Peptidated RNA World spanned ~0.6 Gy (3.6–3.0 Gya), and Protein World’s span amounts to ~3.0 Gy. Thus, the earliest protein folds and domains originated from RNA peptidation eons prior to the appearance of post-PTC ribosome-based protein synthesis. The longer duration of Peptidated RNA World than RNA World is not surprising in view of the introduction of mRNA, tRNA and numerous protein folds and domains, all during the Peptidated RNA World of Stage 4, which amounted to an indispensable inheritance for the Protein World of Stages 5–7.

### 11.3. Paralogs from Code Expansion

In CET, pretran synthesis enabled the conversion of a [precursor aa]-tRNA compound to [product aa]-tRNA, handing to the product amino acid a tRNA and its anticodon that belonged to the precursor amino acid. In so doing, there is a significant probability that the tRNAs of the product and the precursor will turn out to be paralogous. CET thus suggests that tRNAs and eARSs are particularly promising sources of paralogs that can be employed for rooting phylogenetic trees in search of a LUCA. Analysis of eARS sequences shows that the %-identity between same-species TrpRS and TyrRS is highest in Archaea, intermediate in Eukarya and lowest in Bacteria, and the ^129^IALGLRE^135^ sequence in TrpRS and the ^98^IALGLDE^104^ sequence in TyrRS from the euryarchaeon Afu are identical in six out of seven positions, indicating that LUCA was phylogenetically closest to the Archaea [23]. However, the high rate of protein sequence evolution, together with horizontal gene transfers involving not only extant but also extinct species, limits the utility of protein paralogs [224]. In contrast, tRNA sequences have evolved at a slower and steadier tempo, and the paralogous relationships between the GNN-, UNN- and CNN-anticodon bearing isoacceptor tRNAs with Δ = 1 or 2 in the ApeLeu, PaeLeu, PfuLeu, PaeGly, MkaLeu and MkaGly codon boxes are unmistakable (Figure 3C). Such extraordinary sequence ultra-conservatism, bringing about a D_allo_ value as low as 0.351 for *Methanopyrus kandleri*, first revealed the *Methanopyrus*-like nature of LUCA in Stage 6 [110], which was the progenitor of Darwinian evolution in Stage 7.

### 11.4. Feedback for Near Perfection

Once the genetic code added the Phase 2 amino acids to the Phase 1 amino acids, there was no further expansion even though in principle the eight 1aa codon boxes in the present-day code could accommodate eight more amino acids by turning into 2aa boxes. The reason is that each entry of a new amino acid, replacing a preexisting amino acid as the assignee of one or more codons, generated replacement noise. As the performance of the proteome including the translation machinery improved with code expansion, the background translation noise was reduced, rendering the replacement noise less acceptable. Accordingly, code expansion would shut down when translation noise was sufficiently low, *viz.* when the encoded amino acid ensemble was so proficient that a translation error of << 1.5% was achieved [96]. It follows that the evolution of the genetic code and protein alphabet was a relentless search for excellence that never ceased until error feedback signaled that near perfect translation was accomplished. Therefore, even though the protein alphabet is a dynamic rather than frozen construct, it appears to be utterly “frozen” only because its phenomenal success has caused it to remain unchanged throughout the ages, more durable than most geological features on Earth, and universally adopted by all living organisms through the conquest of LUCA’s descendants over competitors equipped with less endowed alphabets [8,160]. This explains the masked but undoubted mutability of the protein alphabet that has made synthetic life possible in Stage 8. The analogy has been drawn that life in all of the past eons, straitjacketed by its protein alphabet of twenty amino acids, may be regarded as the first installment of life, to which the sequel now begins with the arrival of synthetic life [225].

In conclusion, the coevolution theory, proposed to explain the structure of the code, has been verified by wide ranging evidence. In addition, it has revealed the importance of amino acid–RNA cooperation, side chain imperative, tRNA paralogs from code expansion, and feedback to ensure near perfection as factors that prove fundamental as well to the delineation of other stages of life’s emergence. As a result, the eight stages of the emergence process are beginning to be definable as a continuous chain of events uninterrupted by abysmal discontinuities. Identification of an Mka-proximal LUCA clarifies the origins of intron and wobble, and recognition of the Peptidated RNA World as the starting point of peptide coding facilitates analysis of the origins of messenger RNA and transfer RNA. Furthermore, based on the extensive changes undergone by the code to provide codons to biosynthetically derived amino acids, the code is predicted by CET to be an intrinsically mutable, not frozen, code. Experimental proof of this prediction through the isolation of optional and mandatory synthetic life forms has opened up a nascent synthetic life biosphere parallel to the canonical biosphere. The synthetic life forms enable the recruitment of a fast growing variety of unnatural NCAAs into proteins comparable to the NCAAs generated by post-translational modifications. In contrast to post-translational modifications which allow only site-specific NCAA insertions, the altered protein alphabets of synthetic life forms allow both proteome-wide and site-specific NCAA insertions, thereby amplifying the scope of new approaches to a deeper understanding of protein chemistry and evolution. That the DNA alphabet is likewise mutable further expands the potential of synthetic life. The possibility thus arises of designing proteins using altered alphabets with novel amino acids to achieve enhanced performance, and implanting the altered alphabets into transgenic organisms for the production of proteins and vaccines with increased efficacy. Consequently, synthetic life forms can be expected to bring with them a harvest of unimagined insights into how life came about in the past, and how it might develop in the future.

## Figures and Tables

**Table 1:** Phase 1-Phase 2 classification of encoded amino acids.

Evidence	Gly	Ala	Ser	Asp	Glu	Val	Leu	Ile	Pro	Thr	Phe	Tyr	Arg	His	Trp	Asn	Gln	Lys	Cys	Met	Ref.
Coevolution theory	1	1	1	1	1	1	1	1	1	1	2	2	2	2	2	2	2	2	2	2	[92]
Irradiated synthesis	+	+	+	+	+	+	+	+	+	+	0	0	0	0	0	0	0	0	NA	NA	[93,94]
Meteorite composition	+	+	+	+	+	+	+	+	+	+	+	+	0	0	0	0	0	0	0	0	[12,95]
Prebiotic synthesis	+	+	+	+	+	+	+	+	+	+	+	+	0	0	+	+	+	0	+	+	[85,86]
Electric discharge synthesis	+	+	+	+	+	+	+	+	+	+	0	0	0	0	0	0	0	0	0	+	[85,86]

“1” and “2” represent Phase 1 and Phase 2, “+” represents presence, “0” absence, and “NA” not applicable. Meteorite composition indicates presence of Phe and Tyr in Reference [95] although not in Reference [12].

**Table 2:** Lines of evidence of LUCA being archaeal (A) or Mka-proximal (M) or both.

Line No.	Type of Evidence *	Evidence for	Reference
1	Alloacceptor tRNA distances	A, M	[110]
2	Initiator-elongator tRNA^Met^ distances	A, M	[111]
3	Anticodon usages	A, M	[113]
4	Aminoacyl-tRNA synthetase distances	A, M	[111]
5	Archaeal root of ValRS	A	[112]
6	Lack of GlnRS in Mka	M	[112,114]
7	Lack of AsnRS in Mka	M	[112,114]
8	Lack of CysRS in Mka	A, M	[112,114]
9	Lack of cytochromes in Mka	M	[112]
10	Early Euryarchaea-Crenarchaea separation	A, M	[115]
11	Mka as deep branching archaeon	M	[115]
12	Primitivity of methanogenesis	A, M	[115,116]
13	Primitivity of anaerobiosis	M	[117,118]
14	Primitivity of hyperthermophily	A, M	[119,120,121,122,123]
15	Primitivity of barophily	M	[124]
16	Primitivity of acidophily	M	[125,126]
17	Use of CO_2_ as electron acceptor	A, M	[127,128]
18	Chemolithotrophy	M	[112]
19	Hydrothermal vents as appropriate home for LUCA	M	[11,129,130]
20	Minimalist regulations	M	[114]
21	tRNA evolution pattern	A	[131]
22	5S rRNA tree	A	[132]
23	Ribonuclease P tree	A	[133]
24	Protein fold tree	A	[134,135]
25	Proteome tree	A	[135]
26	Slow segregation of Asp and Glu tRNAs	A	Figure 4 and Figure S2
27	Ser tRNA missing link	A, M	Figure 5 and Figure S3
28	Gene ontology	A	[136]
29	Simplistic nucleoside modifications	A	[137,138]

* See reference [8,112].

**Table 3:** Stages of Evolution of Wobble Rules.

Stage	Anticodon *	3rd Codon Base Read	Main Users
I	UNN	U, C, A, G	Pre-LUCA organisms
II	GNN UNN	U, C A, G	Primitive Archaea
III	GNN UNN CNN	U, C A, G G	Majority Archaea
IV	GNN UNN CNN INN	U, C A, G G U, C, A	Bacteria, Eukarya
V	UNN	U, C, A, G in 1aa boxes	Mitochondria, chloroplasts, *Mycoplasma*, *Stretoococcus*, *Borrelia*, *Lactococcus*, *etc.*

* Codons read by anticodons are based on reference [161,164]. Nucleoside modifications that influence wobble occur at codon positions 34 and 35, as well as positions 32, 33, 37–39 of the anticodon loop [162,165,166,167,168,169,170,171]. Modifications of first-base U in UNN anticodons that could restrict wobble are found in both Stage III and Stage IV wobbles, and Um has been identified from the tRNAs of Stage II wobble-user Mka [137].

**Table 4:** Synthetic life systems.

Type *	Insertion	Altered Site	System	Ref.
*o*-Synthetic	NCAA	Proteome-wide	*B. subtilis* LC33, LC88, *E.coli* B7-3: 4FTrp, 5FTrp, 6FTrp; *E. coli* MT16-20: [3,2]Tpa	[178,180,186,187,188,189]
*m*-Synthetic	NCAA	Proteome-wide	*B. subtilis* HR15, HR23: 4FTrp	[178,180]
*o*-Synthetic	NCAA	Specific sites	*E. coli, C. elegans etc.*: *p*-aminoPhe, *p*-azidoPhe *etc.*	[191,192,193,194,195,196,197,198,199,200,201,202,203,204,205,206,207,208]
*m*-Synthetic	NCAA	Specific sites	*E. coli C321*Δ*A*: biphenylPhe *etc.*; *E. coli thyA* R126L: azaLeu	[209,210,211]
*o*-Synthetic	NCDN	Genome-wide	*E.coli* CLU5: 5-chloroU	[212,214]
*m*-Synthetic	NCDN	Genome-wide	*E. coli* CLU5 variant: 5-chloroU	[213]

* *o*-synthetic = optional synthetic; *m*-synthetic = mandatory synthetic.

## Supplementary Materials

The following are available online at http://www.mdpi.com/2075-1729/6/1/12/s1: Figure S1. Gene trees for tRNAs in 2aa codon boxes bearing GNN and UNN anticodons. Figure S2. Sequence alignments of AspGAC and GluGAA genes from different species. Figure S3. Sequence alignments of tRNA genes for Ser from different species.
