# Supplementary material for: Coevolution Theory of the Genetic Code at Age Forty: Pathway to Translation and Synthetic Life
Source: Life (Basel). 2016 Mar 16;6(1):12. doi: 10.3390/life6010012 (PMC4810243; doi:10.3390/life6010012)
Supplement: Supplementary file 1 [file life-06-00012-s001.pdf]

Supplementary Materials: Coevolution Theory of the Genetic Code at Age Forty: Pathway to Translation and Synthetic Life

J. Tze-Fei Wong, Siu-Kin Ng, Wai-Kin Mat, Taobo Hu and Hong Xue

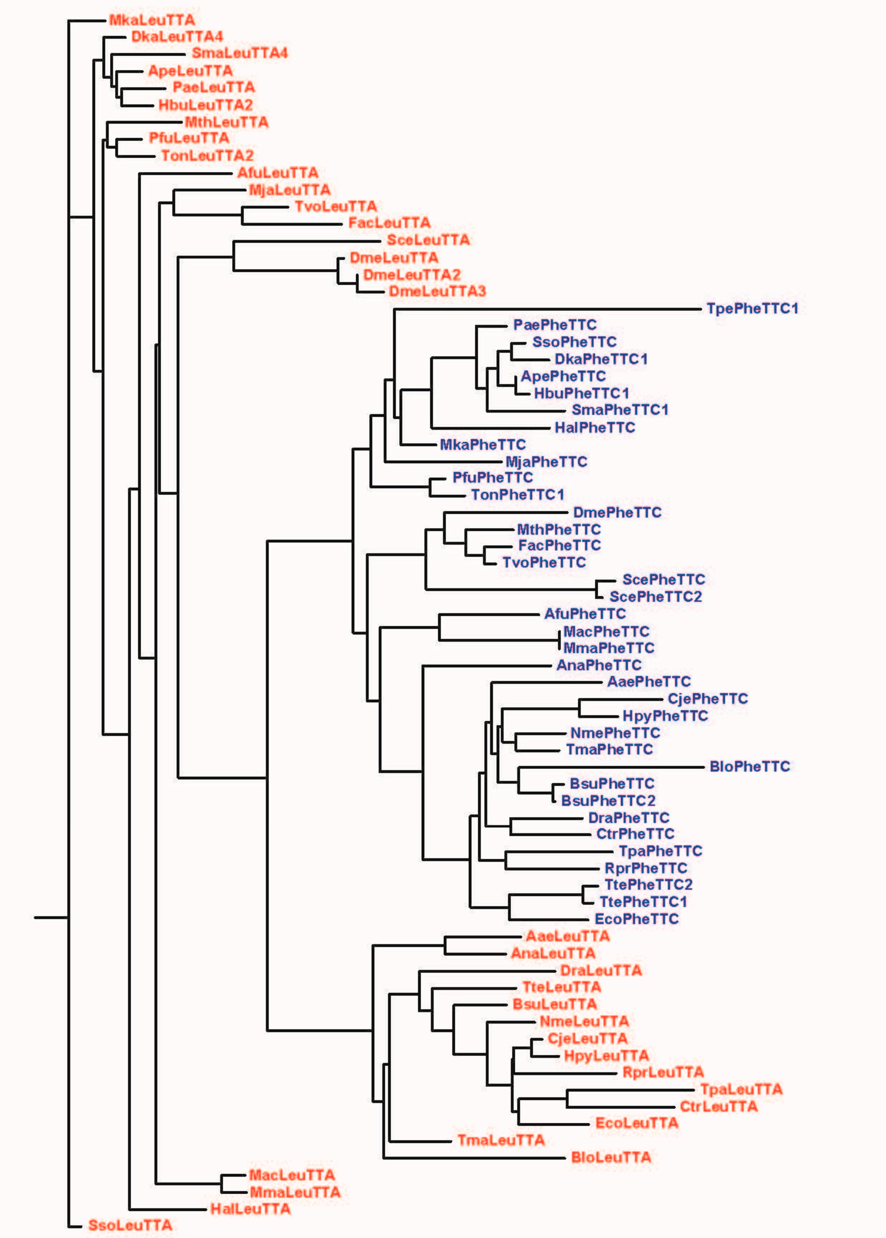

(**A**)

Figure S1. Cont.

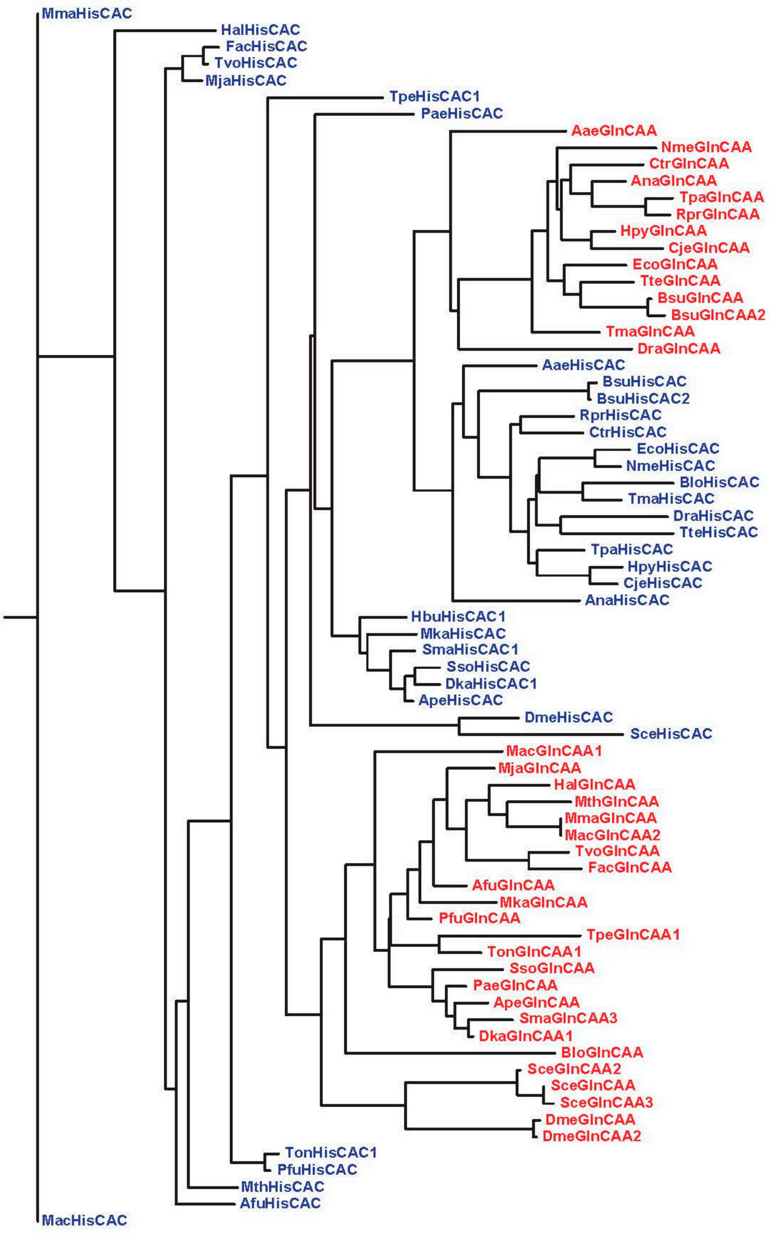

(**B**)

Figure S1. *Cont.*

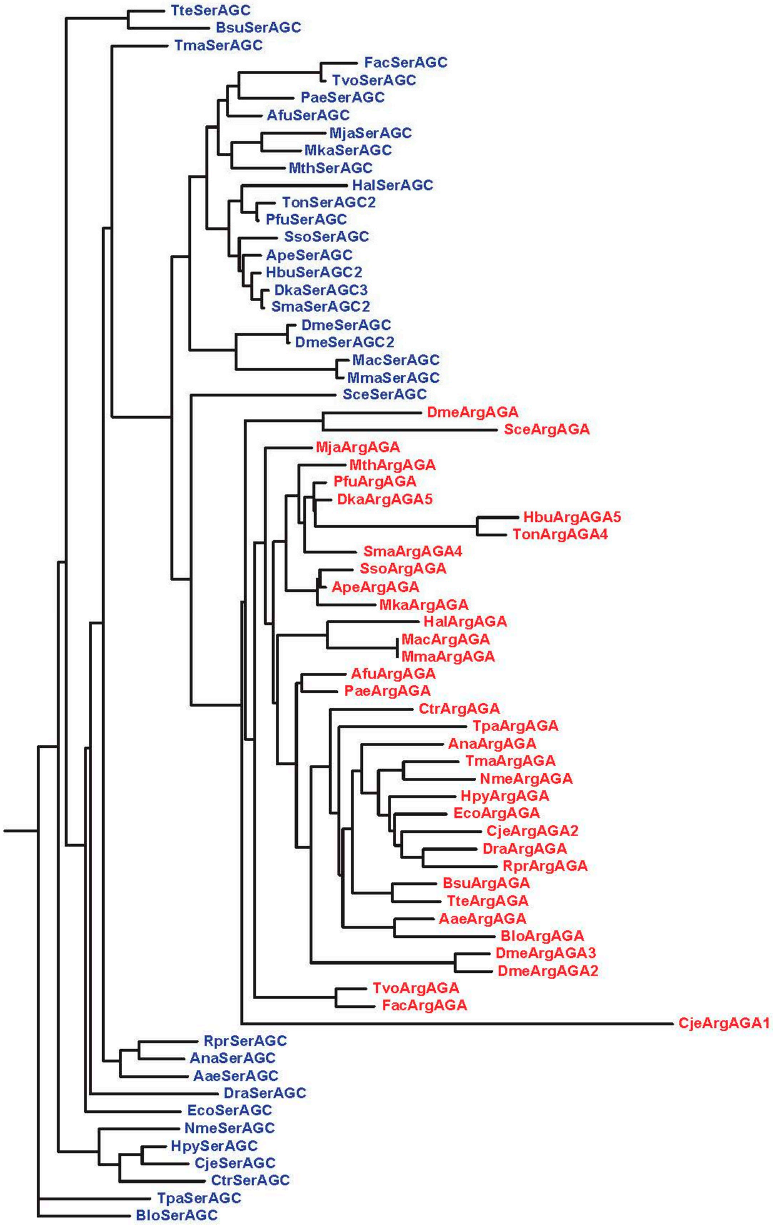

(**C**)

Figure S1. Gene trees for tRNAs bearing GNN and UNN anticodons. (**A**) UUN codon box for Phe/Leu; (**B**) CAN codon box for His/Gln; and (**C**) AGN codon box for Ser/Arg.

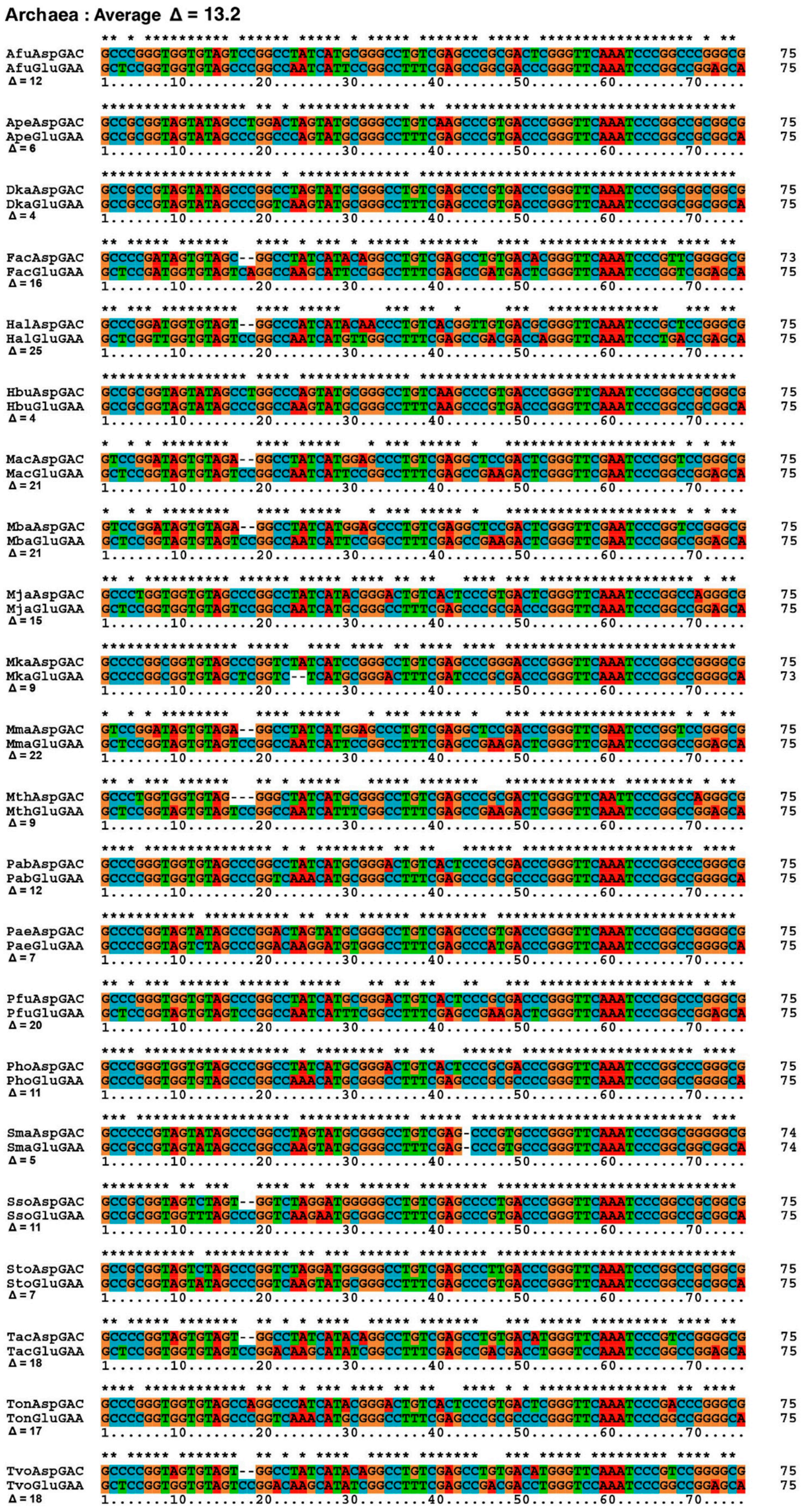

(**A**)

Figure S2. *Cont.*

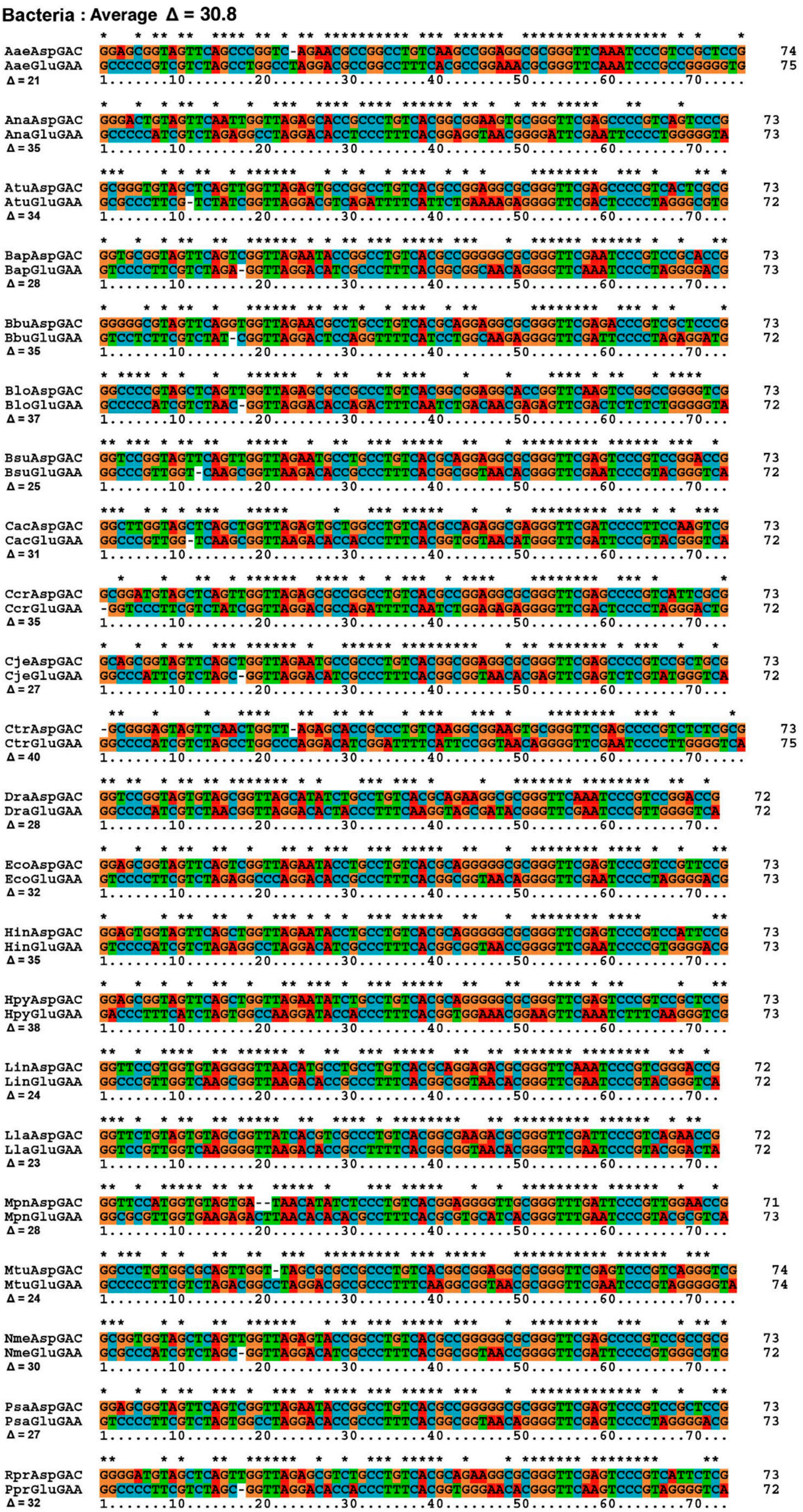

(**A**)

Figure S2. *Cont.*

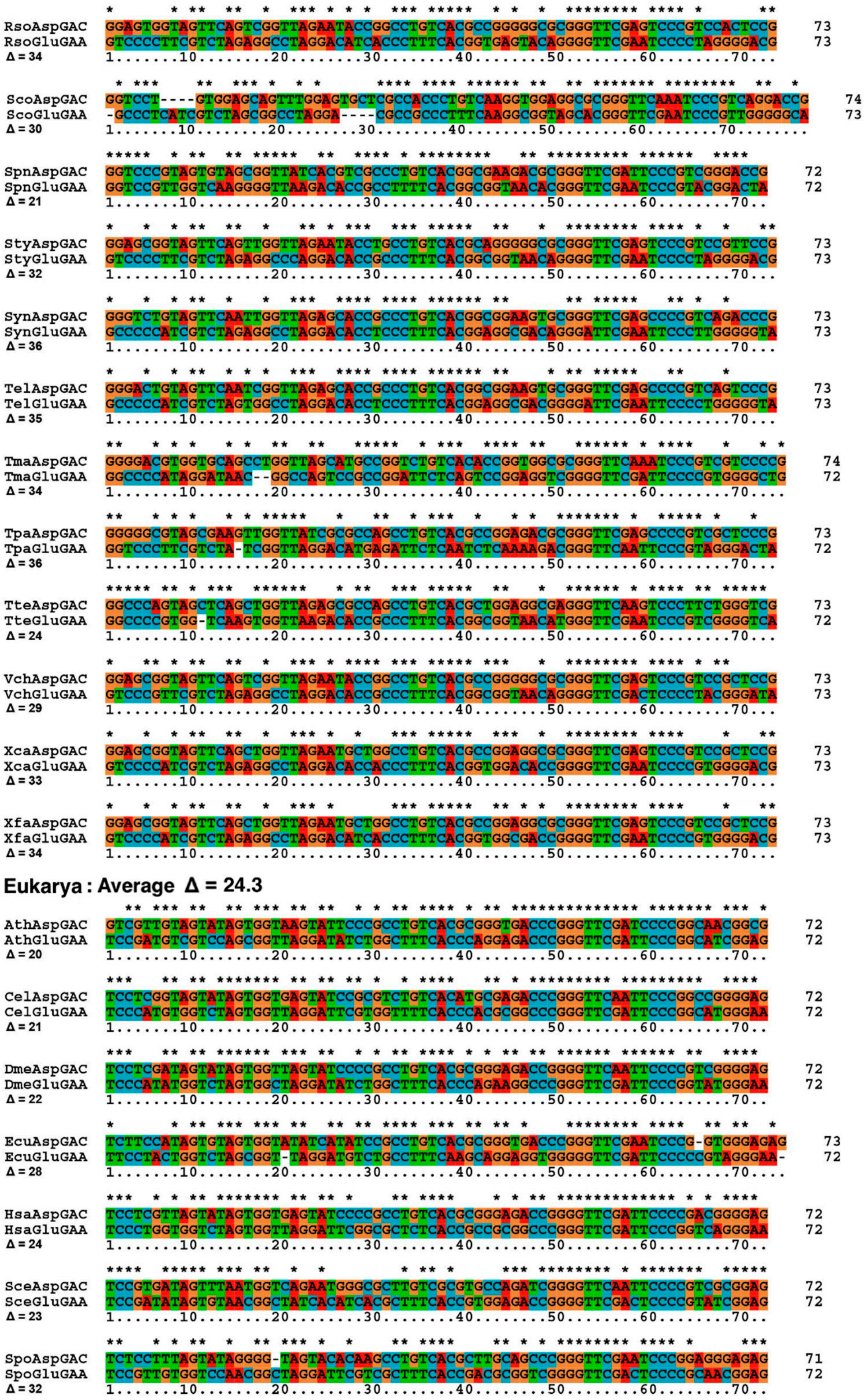

(**A**)

Figure S2. *Cont.*

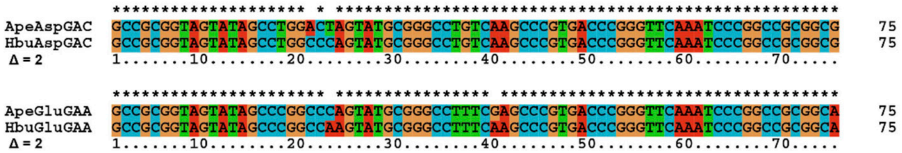

(**B**)

Figure S2. Sequence alignments of AspGAC and GluGAA genes from different species. (**A**) Sequence alignments from different species. The archaeal, bacterial and eukaryotic species aligned comprise species from Figure 4 and reference [83]. (**B**) Sequence alignments between ApeAspGAC and HbuAspGAC, and between ApeGluGAA and HbuGluGAA. Δ represents number of base differences between a pair of aligned genes. Three-letter names for species additional to those included in Figure 4 are: **ARCHAEA.** Mba *Methanosarcina barkeri*, Pab *Pyrococcus abyssi*, Pho *Pyrococcus horikoshii*, Sto *Sulfolobus tokodaii*, Tac *Thermoplasma acidophilum*. **BACTERIA.** Atu *Agrobacterium tumefaciens*, Bap *Buchnera aphidicola*, Bbu *Borrelia burgdorferi*, Cac *Clostridium acetobutylicum*, Ccr *Caulobacter crescentus*, Hin *Haemophilus influenzae*, Lin *Listeria innocua*, Lla *Lactococcus lactis*, Mpn *Mycoplasma pneumoniae*, Mtu *Mycobacterium tuberculosis*, Psa *Pseudomonas aeruginosa*, Rso *Ralstonia solanacearum*, Sco *Streptomyces coelicolor*, Spn *Streptococcus pneumoniae*, Sty *Salmonella typhi*, Syn *Synechocystis 6803*, Tel *Thermosynechococcus elongatus*, Vch *Vibrio cholerae*, Xca *Xanthomonas campestris*, Xfa *Xylella fastidios*. **EUKARYA.** Ath *Arabidopsis thaliana*, Cel *Caenorhabditis elegans*, Ecu *Encephalitozoon cuniculi*, Hsa *Homo sapiens*, Spo *Schizosaccharomyces pombe*.

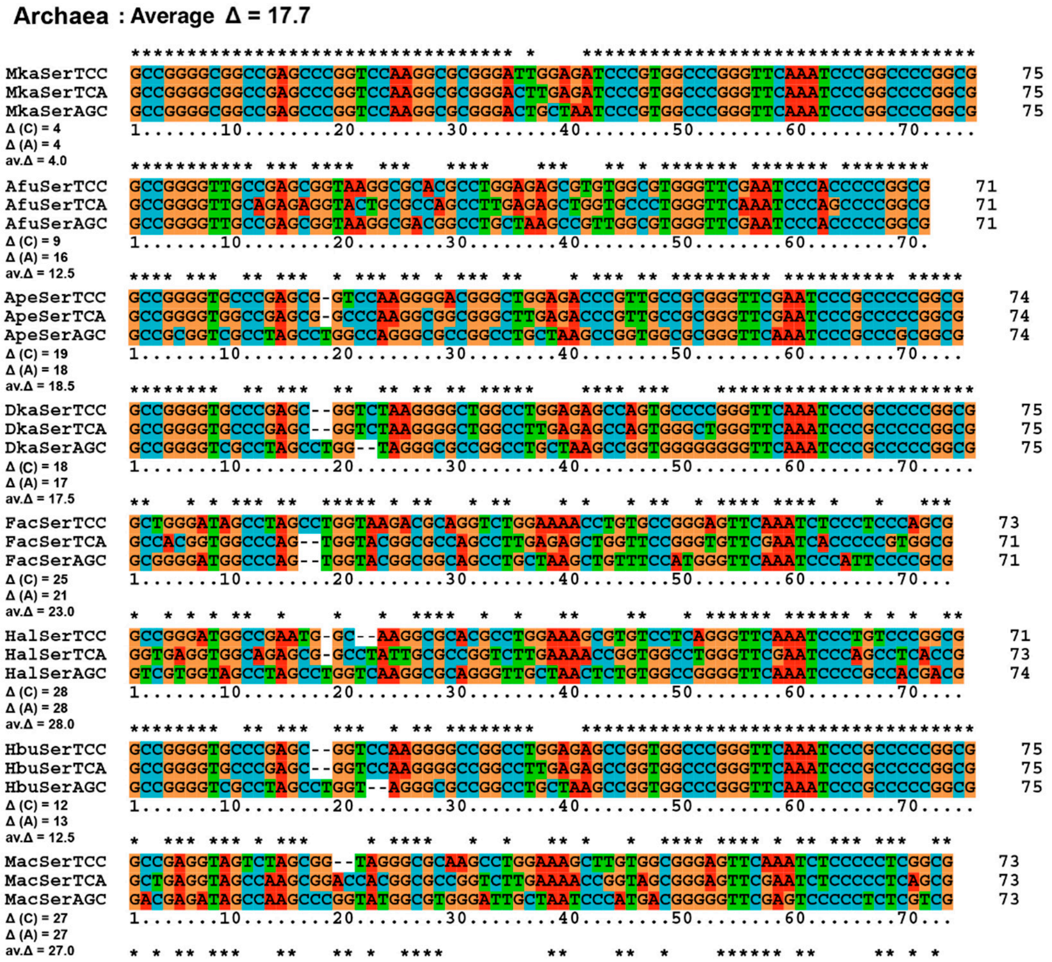

Figure S3. *Cont.*

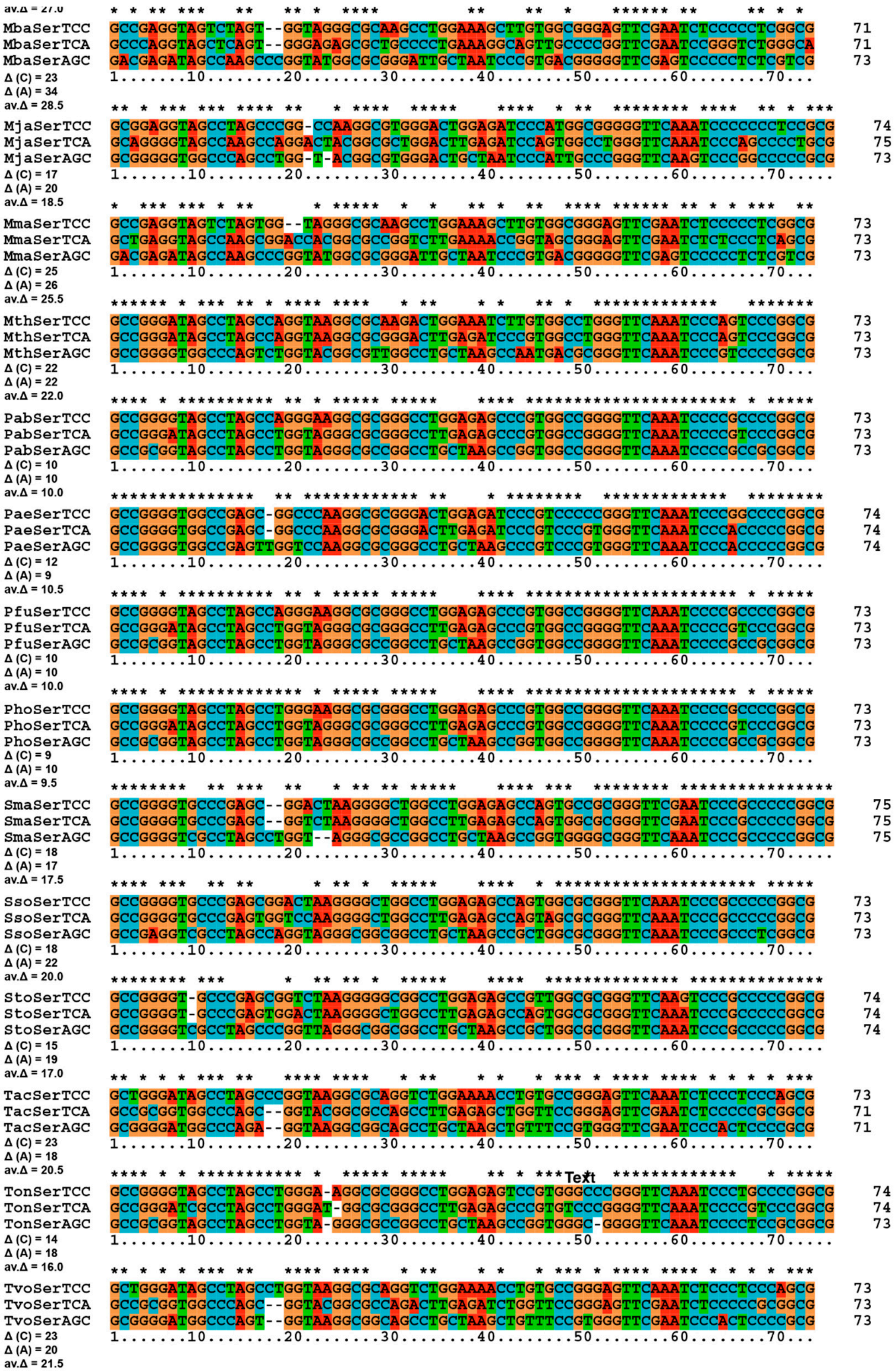

Figure S3. *Cont.*

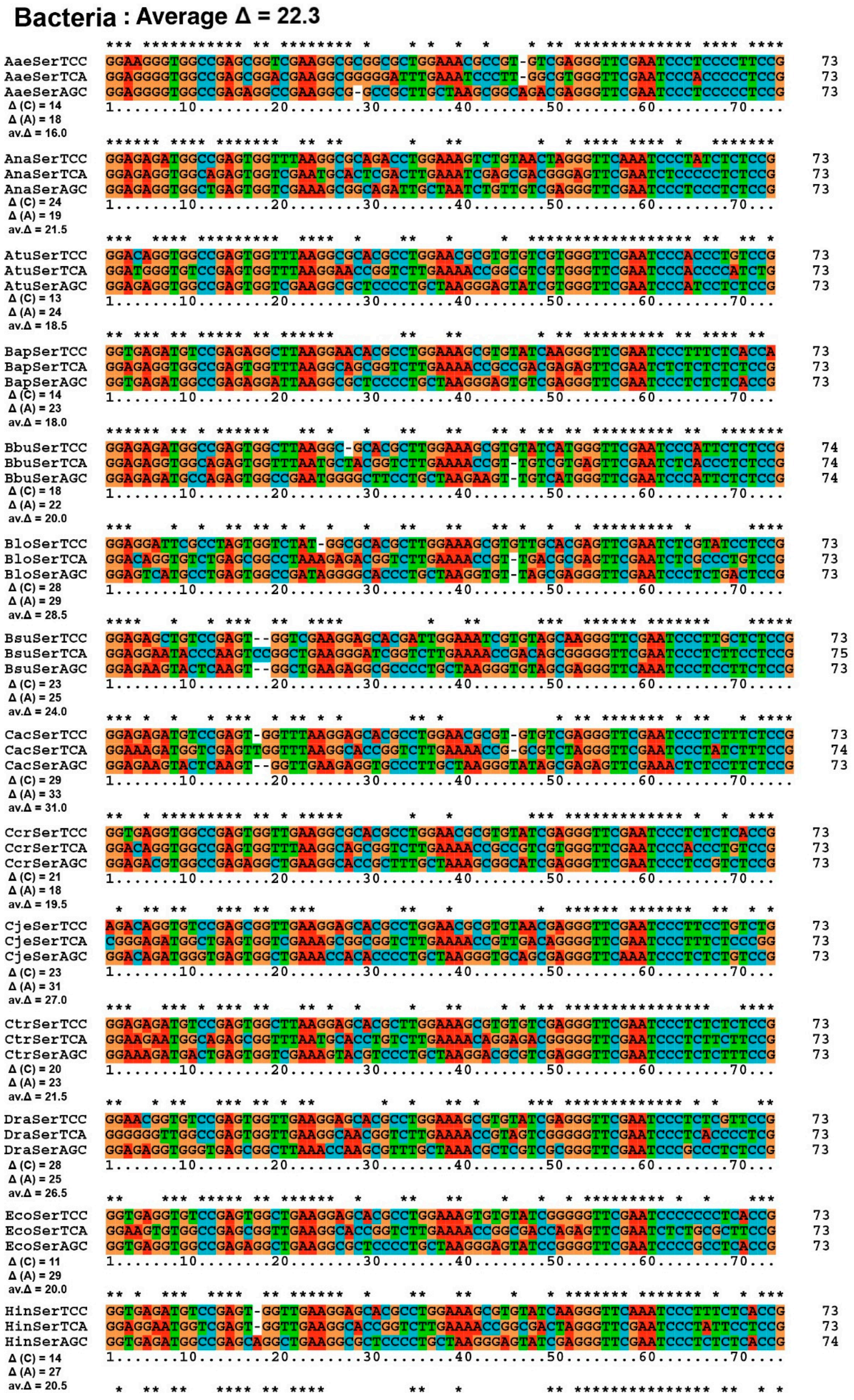

Figure S3. *Cont.*

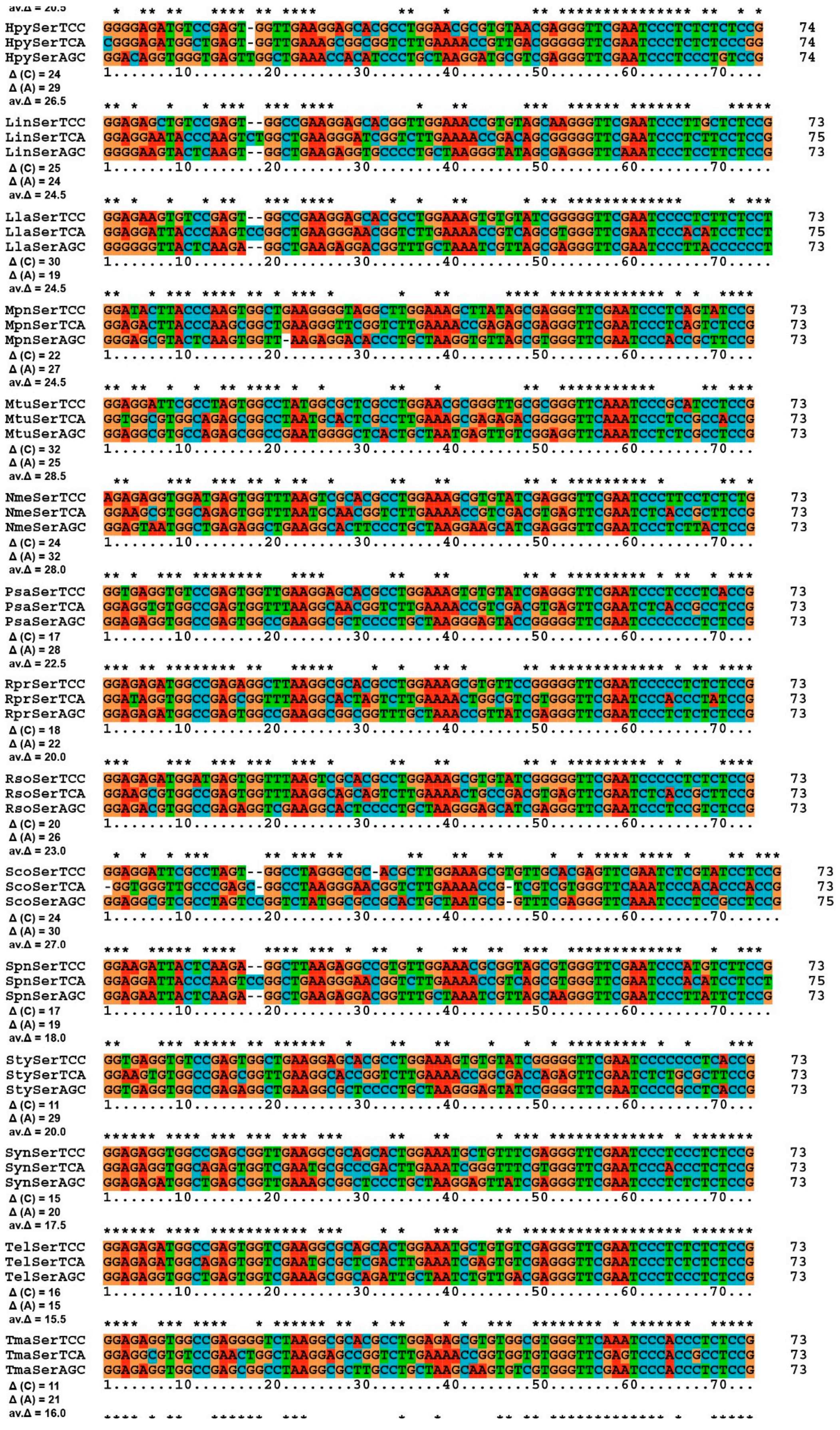

Figure S3. *Cont.*

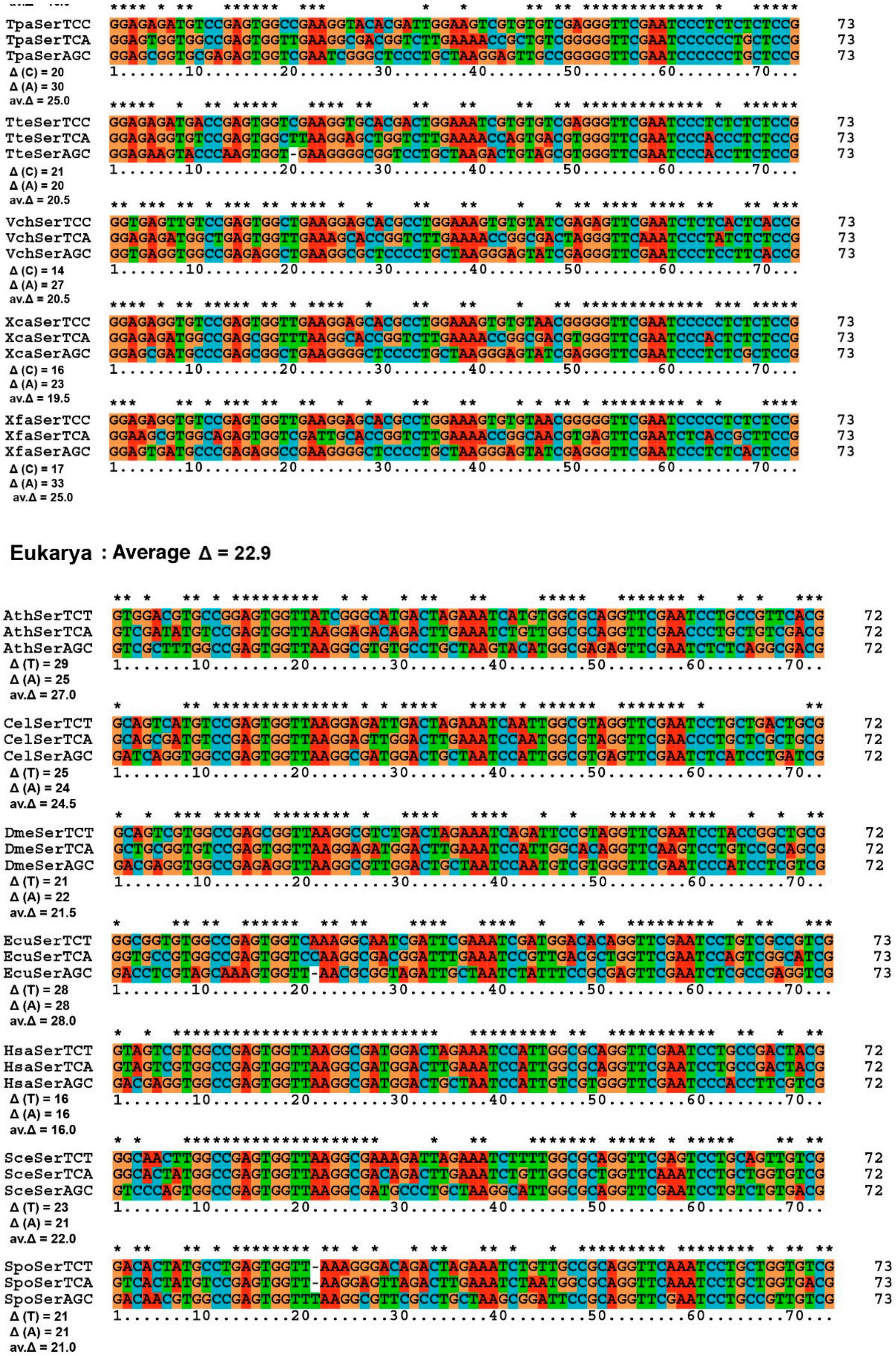

Figure S3. Sequence alignments of tRNA genes for Ser from different species. Δ(C) represents number of base differences between SerAGC and SerTCC, and Δ(A) represents number of base differences between SerAGC and SerTCA, within the same species. Because the eukaryotic species examined lack SerTCC but contain SerTCT, the base difference between SerAGC and SerTCT is estimated instead and shown as Δ(T).
